# Phylogenomic analyses and reclassification of the *Mesorhizobium* complex: proposal for 9 novel genera and reclassification of 15 species

**DOI:** 10.1186/s12864-024-10333-y

**Published:** 2024-04-29

**Authors:** Yan Li, Tingyan Guo, Liqin Sun, En-Tao Wang, J. Peter W. Young, Chang-Fu Tian

**Affiliations:** 1 Yantai Key Laboratory of Characteristic Agricultural Biological Resources Conservation and Germplasm Innovation Utilization, Jiaodong Microbial Resource Center of Yantai University, College of Life Sciences, Yantai University, Yantai, 264005 Shandong China; 2 Departamento de Microbiología, Escuela Nacional de Ciencias Biológicas, Instituto Politécnico Nacional, Ciudad de México, 11340 México; 3 State Key Laboratory of Plant Environmental Resilience, MOA Key Laboratory of Soil Microbiology, Rhizobium Research Center, College of Biological Sciences, China Agricultural University, Beijing, 100193 China; 4 Department of Biology, University of York, York, YO10 5DD UK

**Keywords:** *Mesorhizobium* complex, Phylogenomic, Overall genome relatedness indices, Taxonomy, Reclassification, Symbiotic nitrogen fixation

## Abstract

**Backgroud:**

The genus *Mesorhizobium* is shown by phylogenomics to be paraphyletic and forms part of a complex that includes the genera *Aminobacter, Aquamicrobium, Pseudaminobacter* and *Tianweitania.* The relationships for type strains belong to these genera need to be carefully re-evaluated.

**Results:**

The relationships of *Mesorhizobium* complex are evaluated based on phylogenomic analyses and overall genome relatedness indices (OGRIs) of 61 type strains. According to the maximum likelihood phylogenetic tree based on concatenated sequences of 539 core proteins and the tree constructed using the bac120 bacterial marker set from Genome Taxonomy Database, 65 type strains were grouped into 9 clusters. Moreover, 10 subclusters were identified based on the OGRIs including average nucleotide identity (ANI), average amino acid identity (AAI) and core-proteome average amino acid identity (cAAI), with AAI and cAAI showing a clear intra- and inter-(sub)cluster gaps of 77.40–80.91% and 83.98–86.16%, respectively. Combined with the phylogenetic trees and OGRIs, the type strains were reclassified into 15 genera. This list includes five defined genera *Mesorhizobium*, *Aquamicrobium*, *Pseudaminobacter*, *Aminobacter*and *Tianweitania*, among which 40/41 *Mesorhizobium* species and one *Aminobacter* species are canonical legume microsymbionts. The other nine (sub)clusters are classified as novel genera. Cluster III, comprising symbiotic *M. alhagi* and *M. camelthorni*, is classified as *Allomesorhizobium* gen. nov. Cluster VI harbored a single symbiotic species *M. albiziae* and is classified as *Neomesorhizobium* gen. nov. The remaining seven non-symbiotic members were proposed as: *Neoaquamicrobium* gen. nov., *Manganibacter* gen. nov., *Ollibium* gen. nov., *Terribium* gen. nov., *Kumtagia* gen. nov., *Borborobacter* gen. nov., *Aerobium* gen. nov.. Furthermore, the genus *Corticibacterium* is restored and two species in Subcluster IX-1 are reclassified as the member of this genus.

**Conclusion:**

The *Mesorhizobium* complex are classified into 15 genera based on phylogenomic analyses and OGRIs of 65 type strains. This study resolved previously non-monophyletic genera in the *Mesorhizobium* complex.

**Supplementary Information:**

The online version contains supplementary material available at 10.1186/s12864-024-10333-y.

## Introduction

The genus *Mesorhizobium* belongs to the family *Phyllobacteriaceae* in the order *Hyphomicrobiales* and the class *Alphaproteobacteria* of the phylum *Pseudomonadota* [1]*.* The genus *Mesorhizobium* was established in 1997 and the name reflects the fact that their growth rate was intermediate between that of the genera *Rhizobium* and *Bradyrhizobium* [2]. Bacteria in the genus *Mesorhizobium* were mainly isolated from root nodules of legume hosts, e.g. those belonging to the genera *Acacia*, *Alhagi*, *Amorpha*, *Astragalus*, *Biserrula*, *Caragana*, *Cicer*, *Mimosa*, *Robinia* and *Sophora* distributed all over the world [2–10]. They are characterized by the formation of root nodules and nitrogen fixation. Thus, species from this genus play important roles in the nitrogen cycle of agriculture, prairie and forestry environments. Some strains have been used as efficient inoculants to enhance legume nitrogen fixation, such as *M. ciceri* bv. *biserrulae* WSM1271 used as the commercial inoculant for pasture legume *Biserrula pelecinus* L. in Australia [11]. Nevertheless, type strains of nine of the other *Mesorhizobium* species were not isolated from legume nodules and they did not present nodulation abilities, i.e. *M. comanense* 3P27G6^T^ from groundwater [12], *M. hankyongi* Gsoil 531^ T^, *M. soli* JCM 19897^ T^, *M. terrae* NIBRBAC000500504^T^ and *M. thiogangeticum* SJ^T^ from soil [13–15], *M. composti* CC-YTH430^T^ from compost [16], *M. ephedrae* 6GN30^T^ from root of *Ephedra przewalskii* [17], *M. sediminum* KCTC 42205^ T^ from deep-sea sediment [18], and *M. microcysteis* MaA-C15^T^ from xenic culture of *Microcystis aeruginosa* [19]. Over the years, extensive work has been carried out on classification of *Mesorhizobium* based on polyphasic taxonomy, leading to the description of 71 (63 validly named) species in this genus, making it the largest genus in the family *Phyllobacteriaceae* (https://www.bacterio.net/genus/mesorhizobium/). Among the published species, 37 (accounting for 58%) of them were published in the last ten years (from 2013 till now).

Since the first description of *Mesorhizobium*, its taxonomy has been continuously revised and improved. According to phylogenetic trees constructed using 16S rRNA gene and housekeeping gene (*recA*) sequences, the genus was reported to be polyphyletic, with species *Mesorhizobium camelthorni* and *Mesorhizobium alhagi* forming a distinct lineage distantly with most *Mesorhizobium* species [1]; while *Mesorhizobium albiziae* and *Mesorhizobium thiogangeticum* formed distinct lineages in the *recA* phylogenetic tree [1]. Similar phylogenomic relationships were also apparent in the genome BLAST distance phylogeny (GBDP) tree constructed using the whole genome sequences, since several *Mesorhizobium* species, including *M. camelthorni*, *M. alhagi* and *M. soli,* were intermixed with species from the genera *Aquamicrobium* and *Pseudaminobacter* [20]. Moreover, these three genera together with *Aminobacter* and *Tianweitania* formed an intricate complex that is causing confusion of the taxonomy in the family *Phyllobacteriaceae* [20]. The genus *Aquamicrobium* was established in 1998 [21]. At the present time, the genus comprises 8 validly published species: *A. aerolatum* [22], *A. aestuarii* [23], *A. ahrensii* [24], *A. defluvii* [21], *A. lusatiense* [22], *A. segne* [24], *A. soli* [25] and *A. terrae* [26], and a not validly named species: “*A. zhengzhouense*” [27]. The type strains of this genus were isolated from diverse habitats e.g. sewage [21], tidal flat [23], wastewater-treatment plant [22], air in a duck shed [22], biofilter for the treatment of animal rendering waste gas [24], and contaminated soils [25, 26]. The genus *Pseudaminobacter* was established in 1999 [28]. It comprises five validly published species: *P. arsenicus* [29], *P. defluvii* [28], *P. granuli* [30], *P. manganicus* [31], and *P. salicylatoxidans* [28] and a not validly named species “*P. soli*" [32]. They were isolated from various aquatic environments e.g. arsenic-rich aquifers [29], sludge [28], wastewater treatment plant [30], and river [28]. The genus *Aminobacter* was established in 1992 [33]. It comprises six defined species: *A. aganoensis* [33], *A. aminovorans* [33], *A. anthyllidis* [34], *A. carboxidus* [20], *A. ciceronei* [35] and *A. niigataensis* [33]. They were isolated from soil [33, 35, 36] and root nodule [34]. The genus *Tianweitania* were established in 2016 [37]. It is composed three species, which were isolated from terrestrial sediment, bark tissue and coastal sand respectively [37–39]. As described above, the available species list of this intricate complex is over-represented by *Mesorhizobium* and it is therefore named as the *Mesorhizobium* complex in this work for simplification. Defined genera should be monophyletic [40], so the taxonomy of the species belonging to the *Mesorhizobium* complex needs to be carefully re-evaluated at the genus level.

While the earlier studies on these bacteria were mainly based upon physiochemical, biochemical and chemotaxonomic features in combination with the phylogenetic analysis of 16S rRNA gene [41], later studies used multilocus sequence analysis (MLSA) based on concatenated sequences of several housekeeping genes, such as *recA*, *atpD*, and *glnII* [42, 43] to define the *Mesorhizobium* species. With the rapid progress in genome sequencing technology, genome sequences for the type strains of 50 *Mesorhizobium* species, three of *Aquamicrobium*, three of *Pseudaminobacter*, six of *Aminobacter*, and three for *Tianweitania* are now available in the public database (https://www.ncbi.nlm.nih.gov/genome/). The development of modern bioinformatics makes genome-based studies a promising approach for delineation of species, genera and even higher ranks of bacteria [44]. The average nucleotide identity (ANI) value threshold 95–96% combining with digital DNA-DNA hybridization threshold (dDDH) value of 70% have been suggested as appropriate criteria for species delineation [45, 46]. At the genus and higher rank levels, phylogenetic trees constructed based on the whole genome sequence provides sufficiently precise phylogenetic relationships for bacteria [47–49], however, there is no agreed standard for genus delineation on the basis of genome similarity. A recent study carried out on 3500 type strain genomes of bacteria and archaea found that the threshold between genera was at a mean ANI of 73.98% (25% quartile, 70.85%; 75% quartile, 76.56%) for specific groups [49]. There is still no clear genus ANI demarcation boundary or estimated genus inflection point for all bacteria [49], and measures based on protein sequences have proved more discriminatory than ANI at the genus level [47, 50]. The average amino acid identity (AAI) has been evaluated for genus delineation, and most bacterial intra-genus AAI values were above 68% [51]. Since the phylogenetic relationships or the similarity between the two genomes could be affected by horizontal gene transfer (HGT) [52], it is more reasonable to use the core-proteome average amino acid identity (cAAI) in genus classification to minimize the impact of HGT [48, 53]. The 86% cAAI threshold had effectively improved the delineation of some species belong to equivocal genera of *Rhizobiaceae* [47, 54].

This work aimed to clarify the phylogenetic relationships within the *Mesorhizobium* complex. All type strains of the defined *Phyllobacteriaceae* species with whole genome sequences available in the public databases were used to compare the phylogenomic relationships, and the overall genome relatedness indices (OGRIs), including ANI, AAI and cAAI, were characterized. Furthermore, the phyletic distribution of key nodulation and nitrogen fixation genes was also analyzed. By combining the above results, taxonomic positions were re-evaluated and modified for some ambiguous *Mesorhizobium* complex species.

## Material and methods

### Genome download and dataset

A complete list of all validly published species in the family *Phyllobacteriaceae* was retrieved from LPSN (https://lpsn.dsmz.de/family/phyllobacteriaceae). One genome sequence from each type strain of the corresponding species was selected and downloaded from GenBank, JGI or GCM (http://gctype.wdcm.org/). For the species with more than one genome sequence, the most complete genome sequence was used. The genome quality was evaluated by CheckM v1.2.1 and the genomes with completeness > 95% and contamination < 5% were deemed competent [55]. The genome characteristics including genome size, G + C%, contig numbers and N50 were analyzed by QUAST v4.0 [56].

### Phylogenomic analyses based on core genes

Genes in each qualified genome sequence were predicted and annotated using Prokka v1.13 [57]. Orthologous clusters (OCs) and the core genome sequences were inferred by OrthoFinder v2.5.4 [58]. The single copy ortholog core protein sequences were selected to perform the following analyses: all proteins were aligned using Mafft version 7.471 [59]; the aligned sequences were trimmed by trimAl v1.4 [60]; and the trimmed sequences were concatenated and the maximum likelihood (ML) tree was reconstructed using the best recommended model with the command ‘-m MFP’ by IQ-TREE 2.0.3, with the bootstrap value of 1000 replicates [61]. *Shinella granuli* DSM 18401^ T^ from *Rhizobiaceae* was selected as an outgroup. The tree was visualized and decorated with iTOL v6 online program [62]. The bac120 marker set of 120 genes was selected from Genome Taxonomy Database (GTDB) to infer the phylogenetic relationships using the GTDB-Tk v2.1.0 [63]. The phylogenetic tree based on 16S rRNA gene sequences was also reconstructed using IQ-TREE 2.0.3 [61], with the best recommended model “TPM3 + I + G4” and the bootstrap value was set as 1000 replications.

### Calculation of overall genome relatedness indices (OGRIs)

ANI values between all genome pairs were calculated by using the orthologous average nucleotide identity tool (OrthoANI, v0.93.1) implemented with the blast +  + algorithm [45]. When ANI was larger than 95%, the corresponding genome pair files were further selected to calculate digital DNA-DNA hybridization (dDDH) values by using the Genome-to-Genome Distance Calculator (GGDC, version 3.0) online (https://ggdc.dsmz.de/ggdc.php#) [46]. Genome ANI values more than 95–96% and dDDH values greater than 70% were used as the threshold to determine that strains belonged to the same species [64]. The AAI was calculated from genome sequences between each pair of type strains using CompareM v0.1.2 [53]. For cAAI calculation, the common shared ortholog genes among the 99 *Phyllobacteriaceae* genomes were defined, then the cAAIs between each pair of type strains were calculated by using CompareM v0.1.2 [48, 53].

### Symbiotic nitrogen fixation prediction

Since nitrogen fixation in symbiosis with legume hosts is a prominent feature of most *Mesorhizobium* species [2, 65], the symbiotic nitrogen fixation abilities for the tested type strains were predicted by the presence of *nod* genes (*nodABC*) and nitrogenase cassette (*nifHDK*) in the genome. The *nodABC* and *nifHDK* sequences were extracted from each genome sequence by BLAST +  + software with BLASTN (E-value 1E-5) program [66]. Strains with both *nod* and *nif* genes were potentially bacteria with symbiotic nitrogen fixation abilities.

## Results and discussion

### Genome characteristics of the *Mesorhizobium* complex

A total of 96 genome sequences of the corresponding *Phyllobacteriaceae* type strains were obtained, 87 were downloaded from GenBank and 9 from GCM (Table S1). The completeness for each genome is greater than 95% and the contamination less than 5% detected by checkM (Table S1), indicating that all the genome sequences meet the requirement [55]. Within the *Mesorhizobium* complex, 65 genomes (6 complete and 59 draft) were obtained, the genome size ranges between 3.64 and 8.58 Mb, the G + C% is 60.06–66.43%, the complete sequence with 1–6 replicons and the draft genomes with 7–493 contigs.

### Phylogenetic analyses for *Mesorhizobium* complex species

As in previous studies mentioned in the introduction [1], the genus *Mesorhizobium* is not monophyletic and is intermingled with *Aquamicrobium* and *Pseudaminobacter* in the phylogenetic trees constructed using either 16S rRNA genes or complete genome sequences. In the phylogenetic tree constructed using 16S rRNA gene sequences, all the *Mesorhizobium* type strains are grouped into 6 clusters (Fig. S1) with low bootstrap values (15/94 nodes < 50%). Cluster A includes 31 *Mesorhizobium* species; Cluster B includes three *Mesorhizobium* species: *M. alhagi*, *M. camelthorni*, *M. terrae* and *Chelativorans multitrophicus*; Cluster C contains 12 *Mesorhizobium* species; Cluster D is composed of *M. sediminum* and *Nitratireductor indicus*; Cluster E contains two *Mesorhizobium* species: *M. soli* and *M. ephedrae*; in Cluster F, *M. composti* intermingles with three *Pseudaminobacter* species including *P. manganicus, P. arsenicus* and *P. salicylatoxidans* (Fig. S1).

**Fig. 1:**
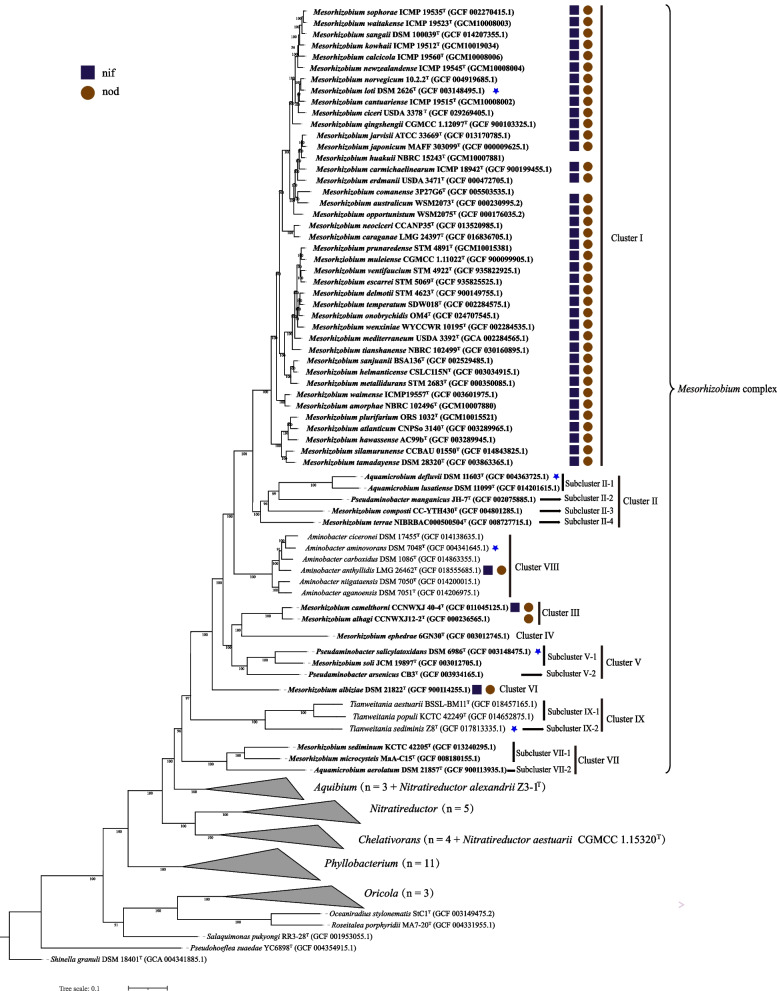
Maximum likelihood (ML) phylogenomic tree based on 539 concatenated core protein sequences of *Phyllobacteriaceae* species with genome sequences. The tree was reconstructed using IQ-TREE 2.0.3 with the best model. Type strains with blue asterisk marks represent type species of the indicated genus. Strain *Shinella granuli* DSM 18401^ T^ was selected as an outgroup

To provide a more robust phylogeny, the phylogenetic relationships among 50 *Mesorhizobium* species with whole genome sequences were evaluated using two distinct sets of core genes (Figs. 1 and S2). A total of 825 single-copy ortholog sequences are shared by the *Mesorhizobium*, *Aminobacter*, *Aquamicrobium*, *Pseudaminobacter* and *Tianweitania* type strains, and 539 are shared by all *Phyllobacteriaceae* type strains and the outgroup strain *Shinella granuli* DSM 18401^ T^ through OrthoFinder analyses. The OrthoFinder ML phylogenetic tree is reconstructed by using the concatenated protein sequence of all the 539 core ortholog genes, in which species belonging to the genera *Mesorhizobium*, *Aminobacter*, *Aquamicrobium*, *Pseudaminobacter* and *Tianweitania* form a *Mesorhizobium* complex and are further reclassified as 9 phylogenomic clusters (Fig. 1) strongly supported by high bootstrap values (58/65 were 100%). The *Mesorhizobium* type strains are grouped into 7 clusters (Cluster I-VII) (Fig. 1). Furthermore, the phylogenetic relationships of the OrthoFinder tree are consistent with the GTDB tree (Fig. S2). Cluster I comprises the type strains representing 41 *Mesorhizobium* species, including the type species *Mesorhizobium loti* [1]. Cluster II consists of two *Mesorhizobium* species (*M. composti* and *M. terrae*), two *Aquamicrobium* species (*A. defluvii* and *A. lusatiense*) and *Pseudaminobacter manganicus*. Cluster III includes two *Mesorhizobium* species (*M. alhagi* and *M. camelthorni*). Cluster V contains the type strains of *Mesorhizobium soli* and two *Pseudaminobacter* species (*P. salicylatoxidans* and *P. arsenicus*). Cluster VII is composed of two *Mesorhizobium* species (*M. microcysteis* and *M. sediminum*) and *Aquamicrobium aerolatum* (Figs. 1 and S2). Clusters IV and VI each cover only a single strain of *Mesorhizobium ephedrae* and *Mesorhizobium albiziae*, respectively. In short, the phylogenetic relationships of both the 16S rRNA gene and the whole genome sequences indicate that *Mesorhizobium* is not monophyletic and the taxonomy of *Mesorhizobium* complex should be re-evaluated.

### Determination of the overall genome relatedness indices among the *Mesorhizobium* complex species

**Fig. 2:**
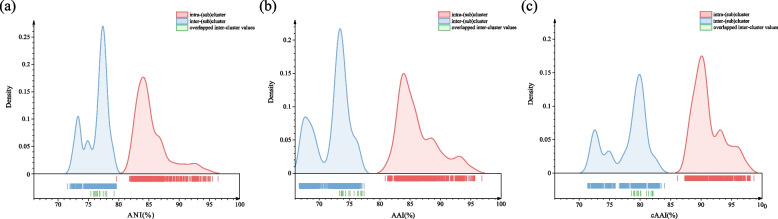
Distribution graphs showing the **a** ANI, **b** AAI and **c** cAAI density generated by pairwise comparisons among all 61 genomes belonging to the *Mesorhizobium* complex. The red lines indicate comparisons between genomes in the same cluster or subcluster, blue lines are for genomes in different (sub)clusters, and green lines are for overlapped data between inter- and intra-clusters

**Fig. 3:**
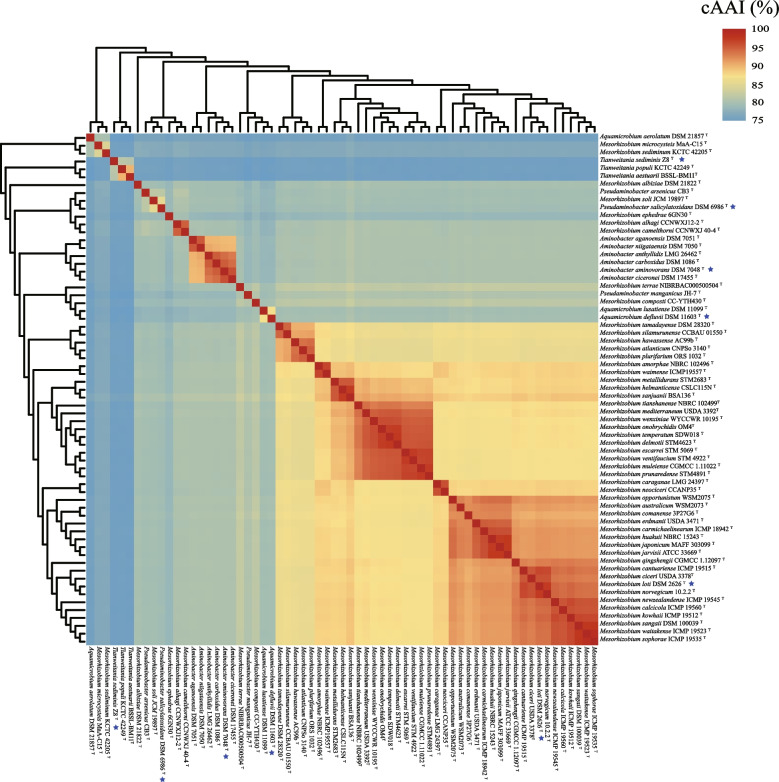
Heatmap depicting the cAAI values between each pair of type strains belong to the *Mesorhizobium* complex classified in this study. Type strains with blue asterisk marks represent type species of the indicated genus

All the type strains belonging to the *Mesorhizobium* complex were selected to compare the genome pair OGRIs. The ANI, AAI and cAAI (using the complex common shared 853 single copy protein sequences) values of the genome pairs are 71.58–96.42%, 66.70–96.85% and 71.41–98.67% respectively (Figs. 2, S3, S4 and S5). Moreover, a notable gap can be found for the each of the OGRIs, i.e. 79.64–81.80% for ANI, 77.40–80.91% AAI and 83.98–86.16% for cAAI (Fig. S3). The inter-cluster and intra-cluster ANI values are 71.58–79.64% and 75.41–96.42% respectively (Figs. 3, S3 and Table S2), while the inter-cluster AAI values range from 66.70% to 77.40%, and the intra-cluster AAI values vary from 73.35% to 96.85% (Fig. S4 and Table S2). The inter-cluster and intra-cluster cAAI values are 71.41–83.98% and 78.59–98.67% (Fig. 2 and Table S2). The overlap between intra-cluster and inter-cluster values is attributable to Clusters II, V, VII and IX, which have intra-cluster values of 75.41–79.52% for ANI, 73.35–77.40% for AAI, and 78.61–82.09% for cAAI (Fig. 3).

Combining the phylogenetic trees and the OGRIs, a total of 10 subclusters in the *Mesorhizobium* complex are further classified within the four clusters (Cluster II, V, VII and IX) (Figs. 1 and S2). Furthermore, the classification of 15 (sub)clusters including 5 undivided clusters (Cluster I, III, IV, VI, and VIII) and 10 subclusters is perfectly in line with the density distribution pattern of OGRIs (Fig. 3). The only exception is the intra-subcluster ANI value of 79.62%, between *M. microcysteis* MaA-C15^T^ and *M. sediminum* KCTC 42205^ T^ which overlaps the inter-(sub)cluster ANI values (Figs. 3 and S3). The other intra-(sub)cluster ANI values are 81.80–96.42%, the intra-(sub)cluster AAI and cAAI values are 80.91–96.85% and 86.16–98.67% respectively (Fig. 3). The 15 inter-(sub)cluster ANI, AAI and cAAI are 71.58–79.64%, 66.70–77.40% and 71.41–83.98% (Fig. 3), respectively. Hence, our study supports previous findings that AAI and cAAI are more suitable to define genus rank than ANI [48, 50, 51]. In our study, all the cAAI values within the 15 (sub)clusters are higher than 86.16%, which is slightly higher than the proposed 86% cAAI threshold for genus delimitation [47], and the inter-(sub)cluster cAAI values are lower than 83.98% (Fig. 3). Thus, each of the 5 independent clusters and 10 subclusters (Figs. 1 and S2) may represent a different genus. The AAI (77.40–80.91%) and cAAI (83.98–86.16%) gaps between intra- and inter-(sub)cluster values also support this reclassification of genera in the *Mesorhizobium* complex.

### Prediction of symbiotic nitrogen fixation abilities for *Mesorhizobium* species

The prominent feature of most species in the genus *Mesorhizobium* is that they nodulate with legume hosts; however, there are *Mesorhizobium* species isolated from soil or even deep-sea sediment [13, 18]. *Mesorhizobium* species in Cluster I, Cluster III and Cluster VI harbored both *nod* and *nif* genes (Fig. 1), consistent with the fact that most of them were isolated from root nodules [3, 67, 68] (Table S3). The few exceptions among them were *M. comanense* 3P27G6^T^ and *M. huakuii* NBRC 15243^ T^ in Cluster I, in which *nod* and *nif* genes are absent. Strain *M. comanense* 3P27G6^T^ was isolated from ground water [12]; while *M. huakuii* NBRC 15243^ T^ was originally isolated from root nodule of *Astragalus sinicus* and its nodulation ability was confirmed when the species was described [2, 69]. Since *nod* genes have been located on the symbiotic plasmids in other strains of this species, such as *M*. *huakuii* 7653R [70], it is possible that the type strain *M*. *huakuii* NBRC 15243^ T^ had lost its symbiotic plasmid during the subculture procedures in laboratory [71]. Another two *Mesorhizobium* strains *M. camelthorni* CCNWXJ 40-4^ T^ (Cluster III) and *M. albiziae* DSM 21822^ T^ (Cluster VI) harbor the complete *nod* and *nif* genes. However, strain *M. alhagi* CCNWXJ12-2^ T^ (Cluster III) possesses only the *nod* genes but not the *nif* genes, which may be due to loss in subculture or defects of sequencing and assembly, since a *nifH* sequence was reported in the original publication [68]. The above results indicate that strains from the clusters I, III and VI possess the potential of symbiotic nitrogen fixation with the corresponding legume hosts. In this study, all the *Mesorhizobium* type strains in Cluster II, IV, V and VII lacked *nod* and *nif* genes, and they were isolated from compost (*M. composti* CC-YTH430^T^) [16], soil (*M. terrae* NIBRBAC000500504^T^) [14], root endosphere (*M. ephedrae* 6GN30^T^) [17], rhizosphere (*Mesorhizobium soli* JCM 19897^ T^) [15], and aquatic environments (*M. sediminum* KCTC 42205^ T^ and *M. microcysteis* MaA-C15^T^) [18, 19].

### Genus and species level reconsiderations for *Mesorhizobium* complex

The combined evidence of the Orthofinder and GTDB phylogenetic trees that are reconstructed using the genome sequences (Figs. 1 and S2) and the intra-(sub) cluster and inter-(sub)cluster OGRIs (Figs. 2, 3, Table 1, Figs. S3, S4) indicates that each of the 5 independent clusters and the 10 subclusters in the *Mesorhizobium* complex defined in the present study merit the rank of a genus. Four legume-nodulating clusters belong to two defined (Cluster I and VIII) and two novel (Cluster III and VI) genera. The 41 species in Cluster I should be maintained as genus *Mesorhizobium*, since the type species *M. loti* represented by strain DSM 2626^ T^ (Fig. 1) is in this cluster. The genome size for the type strains in this cluster varies from 6.20 to 8.58 Mb; the G + C content of genome DNA is between 61.84% and 64.00%; the intra-cluster ANI, AAI and cAAI are 81.80–96.42%, 81.29–96.85% and 88.18–98.67%, respectively (Table 1, Figs. 2, and S4), which are clearly above the gap between intra- and inter-genus values. In this cluster (genus), the ANI and dDDH between *M. sophorae* ICMP 19535^ T^ and *M. waitakense* ICMP 19523^ T^ are 96.42% and 72.2% (Fig. S3 and Table S4), which exceed the species threshold of 95–96% and 70% [45, 46, 72], respectively. Based on the facts that both species were isolated from nodules of *Sophora microphylla* and could nodulate with it [10], and they presented very similar genome size, G + C% (Table S1), physiochemical characteristics, major fatty acids and symbiotic phenotypes [10], they should be combined into a single species. They were published simultaneously, but the species name *M. sophorae* would be preferred for the combined species (reflecting the original host). Although the ANI values between three species pairs of *M. escarrei* STM 5069^ T^ and *M. ventifaucium* STM 4922^ T^, *M. delmotii* STM 4623^ T^ and *M. temperatum* SDW018^T^, *M. delmotii* STM 4623^ T^ and *M. onobrychidis* OM4^T^ are greater than 95% (95.02–95.52%), the DDH values between each pair were less than 70% (62.50–64.30%) (Table S4), thus they can be maintained as independent, but closely related species, like some species in the *Rhizobium leguminosarum* complex [73]. The 6 species in Cluster VIII should be maintained as genus *Aminobacter*, since the type species *Aminobacter aminovorans* represented by strain DSM 7048^ T^ (Figs. 1 and S2) is in this cluster. The genome size for the type strains in this cluster varies from 5.29 to 6.78 Mb; the G + C content of genome DNA is between 62.58% and 63.89%; the intra-cluster ANI, AAI and cAAI are 85.21–91.11%, 86.75–93.93% and 91.81–96.55% respectively (Table 1, Figs. 2, and S4), which are clearly above the gap between intra- and inter-genus values. Strain *Aminobacter anthyllidis* LMG 26462^ T^ is the only nodulating bacterium with both *nod* and *nif* related genes in the cluster [34]. Cluster III includes two symbiotic *Mesorhizobium* species *M. alhagi* and *M. camelthorni* possessing *nod* and *nif* genes, both were isolated from root nodules of *Alhagi sparsifolia* and could nodulate with their original host [3, 68] (Table S3). Their genome sizes are 6.97 and 7.3 Mb, with DNA G + C% 62.65 and 62.41%, the intra-cluster ANI, AAI and cAAI are 91.13%, 91.75% and 96.04%, respectively (Fig. 1 and Table S1). As an independent cluster in both phylogenetic trees, Cluster VI included only *M. albiziae* DSM 21822^ T^ that is a symbiont of *Albizia kalkora* [67], harboring both *nod* and *nif* genes (Fig. 1). It has a genome size of 6.27 Mb and DNA G + C% of 62.08%. Clusters III and VI represent novel genera, as they do not include any type species, but the type strains had canonical symbiotic ability like those of Cluster I species (*Mesorhizobium*), so we propose the names *Allomesorhizobium* and *Neomesorhizobium* for the two novel genera.

**Table 1:** Genomic information and OGRIs of the *Mesorhizobium* complex

(Sub)Cluster	Genome size (Mb)	G + C(%)	Intra-(sub)cluster ANI (%)	Intra-(sub)cluster AAI (%)	Intra-(sub)cluster cAAI (%)
Cluster I	6.20–8.58	61.84–64.00	81.80–96.42	81.29–96.85	87.32–98.67
Cluster II	4.39–6.02	61.20–63-17	75.97–84.14	73.35–86.77	78.61–89.61
Subcluster II-1	4.39–4.52	62.60–63.15	84.14	86.77	89.61
Subcluster II-2	4.65	65.18	–	–	–
Subcluster II-3	4.84	61.20	–	–	–
Subcluster II-4	6.02	63.17	–	–	–
Cluster III	6.97–7.30	62.41–62.65	91.13	91.75	95.80
Cluster IV	6.11	66.43	–	–	–
Cluster V	4.84–6.27	61.42–62.68	77.79–82.70	76.36–82.96	81.98–87.68
Subcluster V-1	4.84–6.27	62.57–62.68	82.70	82.96	87.68
Subcluster V-2	5.21	61.42	–	–	–
Cluster VI	6.27	62.08	–	–	–
Cluster VII	3.64–6.14	60.06–64.14	75.41–79.62	74.90–80.91	79.72–86.16
Subcluster VII-1	4.84–6.14	63.27–64.14	79.62	80.91	86.16
Subcluster VII-2	3.64	60.06	–	–	–
Cluster VIII	5.29–6.78	62.58–63.89	85.21–91.11	86.75–93.93	91.85–96.56
Cluster IX	3.83–4.70	61.30–61.83	76.29–84.71	76.85–88.82	81.34–92.14
Cluster IX-1	3.83–4.29	61.30–61.39	84.71	88.82	92.14
Cluster IX-2	4.70	61.83	–	–	–

The other 11 members with neither *nod* nor *nif* genes belong to four defined genera (Subcluster II-1, Subcluster V-1, Sucluster IX-1 and Subcluster IX-2) and seven novel genera (Cluster IV, Subclusters II-2, 3, 4, Subcluser V-2, Subcluser VII-1, 2). Subcluster II-1 should be maintained as genus *Aquamicrobium*, since the type species *Aquamicrobium defluvii* represented by strain DSM 11603^ T^ is included in the subcluster; it is composed of two *Aquamicrobium* species (*A. defluvii* and *A. lusatiense*) (Figs. 1, and S2, Table 1). Their genome sizes are 4.39 and 4.52 Mb, with G + C content of 62.60% and 63.15%, and the ranges of values of intra-cluster ANI, AAI and cAAI are 84.14%, 86.77% and 89.61%, which are clearly higher than the gap between intra- and inter-genus values (Figs. 2, S3, S4 and Table 1). Both type strains were isolated from sludge (Table S3) [21]. Subcluster V-1 includes *Pseudaminobacter salicylatoxidans* and *Mesorhizobium soli* (Figs. 1 and S2). Their genome sizes are 4.84 and 6.27 Mb, with DNA G + C% 62.68% and 62.57%, and their intra-subcluster ANI, AAI and cAAI are 82.7%, 82.96% and 87.50%, respectively (Figs. 2, S3, and S4), which are also higher than the gap between intra- and inter-genus values. They were isolated from river [28] and rhizosphere [15] (Table S3). Since *Pseudaminobacter salicylatoxidans* DSM 6986^ T^ represented the only type species in this subcluster, both species in this cluster should be reclassified as members of the genus *Pseudaminobacter*. Cluster IX including three *Tianweitania* strains, they are further classified into two subclusters. The AAI and cAAI intra-Subcluster IX-1 is 88.82% and 92.14%, which obviously higher than the gap between intra- and inter-genus values (Figs. 2, S3 and S4 and Table 1). But the AAI and cAAI between Subcluster IX-2 (*T. sediminis*) and Subcluster IX-1 are: 76.55% and 76.30%, 77.09% and 76.85% respectively, which are clearly lower than the gap between intra- and inter-genus values (Figs. 2, S3 and S4 and Table 1). Combined with the substrate utilization characteristics [39] and ORGIs of our study, the Cluster IX class into two genera is reasonable. Subcluster IX-1 includes *T. aestuarii and T. populi* (Figs. 1 and S2), their genome sizes are 3.83 and 4.29 Mb, they were isolated from bark tissue and coastal sand (Table S3). For *T. populi* is the type species of previos *Corticibacterium*, and the genus name should be restored and the both strains should be reclassified as *Corticibacterium*. And the species *T. sediminis* in Subcluster IX-2 should be maintained as *Tianweitania*. Subcluster VII-1 includes two former *Mesorhizobium* species, *M. sediminum* KCTC 42205^ T^ and *M. microcysteis* MaA-C15^T^ (Figs. 1 and S2). Their genome sizes are 6.14 and 4.84 Mb, with G + C% 63.27% and 64.14%, and with AAI and cAAI values of 80.91% and 85.99% between them, respectively (Table 1, Fig. S3 and S4), which are clearly higher than the gaps between intra- and inter-genus values. Thus, they should be classified as the same genus. They were isolated from aquatic environments, including xenic culture of *Microcystis aeruginosa* [19] and sediment [18] (Table S3). As there is no type species in this subcluster and both strains were isolated from aquatic environments, we propose *Neoaquamicrobium* as the name for this novel genus.

Each of the other six (sub)clusters, Clusters IV, Subclusters II-2, 3, 4, V-2 and VII-2, includes only one species, and each forms an independent branch in the phylogenomic trees (Figs. 1 and S2). The OGRIs between these type strains and other type strains belong to the *Mesorhizobium* complex are lower than the gap between intra- and inter-genus values (Figs. 2,  S3 and S4). As none of these species is the type species of a genus, it is reasonable to reclassify each of them as a new genus. For Subcluster II-2, *Pseudaminobacter manganicus* JH-7^ T^ was isolated from sludge of a manganese mine [31], and we propose *Manganibacter* as the genus name. For Subcluster II-3, *Mesorhizobium composti* CC-YTH430^T^ was isolated from compost and lacks *nod* and *nif* genes [16], so a name based on *Mesorhizobium* would be inappropriate and we propose *Ollibium* as the genus name. For Subcluster II-4, *Mesorhizobium terrae* NIBRBAC000500504^T^ was isolated from soil and lacks *nod* and *nif* genes [14], and we propose *Terribium* as the genus name. For Cluster IV, *Mesorhizobium ephedrae* 6GN30^T^ was isolated from root endosphere of *Ephedra przewalskii* and lacks *nod* and *nif* genes [17], and we propose *Kumtagia* as the genus name. For Subcluster V-2, *Pseudaminobacter arsenicus* CB3^T^ was isolated from arsenic-rich aquifers [29], and we propose *Borborobacter* as the genus name. For Subcluster VII-2, *Aquamicrobium aerolatum* DSM 21857^ T^ was isolated from air sampled in a duck shed [22], thus we propose *Aerobium* as the genus name.

In conclusion, the taxonomy of species in *Mesorhizobium* complex should be revised based upon the analyses of whole genome sequences. Both the phylogenomic results and OGRIs support the division of the species belonging to the complex into 15 genera including 5 defined (Cluster I, Subcluster II-1, Subcluster V-1, Cluster VIII and Cluster IX-2 corresponding to *Mesorhizobium*, *Aquamicrobium, Pseudaminobacter*, *Aminobacter* and *Tianweitania*) and 9 novel genera (Cluster III, IV, VI, Subclusters II-2, 3, 4, V-2, VII-1, VII-2). Clusters I, III, VI and VIII include symbiotic strains harboring both *nod* and *nif* genes.

### Taxonomic consequences

1. Description of *Manganibacter* gen. nov.

*Manganibacter* (Man.ga.ni.bac′ter, N.L. neut. n. *manganicum*, manganese; N.L. masc. n. *bacter*, rod; N.L. masc. n. *Manganibacter*, a rod-shaped bacterium isolated from a manganese mine).

Cells are Gram-stain-negative, anaerobic, non-motile, capsule-forming and rod-shaped bacterium. The genus represents a distinct branch in the family *Phyllobacteriaceae* of the class *Alphaproteobacteria* based on the core-genomic ML phylogeny. The genome size of the type strain is 4.84 Mb and the DNA G + C content is 61.2%. The type species is *Manganibacter manganicus*.

**Description of Manganibacter manganicus comb. nov.**

*Manganibacter manganicus* (man.ga′ni.cus. N.L. masc. adj. *manganicus*, referring to its association with a manganese mine).

Basonym: *Pseudaminobacter manganicus* Li et al*.* 2017.

The description is the same as *P. manganicus* [31]. The type strain is JH-7^ T^ (= KCTC 52258^ T^ = CCTCC AB 2016107^ T^) isolated from sludge of a manganese mine near Tongren city, Guizhou Province of China. The DNA genome size is 4.84 Mb, the G + C content of the type strain is 61.2% (by genome).

2. Description of Ollibium gen. nov.

*Ollibium* (Ol.li′bi.um, L. fem. n. *olla*, plant pot; Gr. masc. n. *bios*, life; N.L. neut. n. *Ollibium*, a bacterium that lives in a plant pot).

Cells are Gram-stain-negative, facultative anaerobic rod-shaped bacterium, that formed yellow-colored colonies on nutrient agar. The genus represents a distinct branch in the family *Phyllobacteriaceae* of the class *Alphaproteobacteria* based on the core-genomic ML phylogeny. The genome size of the type strain is 4.65 Mb and the DNA G + C content is 65.18%. The type species is *Ollibium composti*.

**Description of Ollibium composti comb. nov.**

*Ollibium composti* (com.posAAti. N.L. gen. n. *composti* of compost).

Basonym: *Mesorhizobium composti* Lin et al*.* 2020.

The description is the same as *M. composti* [16]. The type strain is CC-YTH430^T^ (= BCRC 81024^ T^ = JCM 31762^ T^), and was isolated from a glasshouse compost sample in Taiwan. The genome size is 4.65 M and the DNA G + C content of the type strain is 65.18% (by genome).

3. Description of Terribium gen. nov.

*Terribium* (Ter.ri′bi.um, L. fem. n. *terra*, soil; Gr. masc. n. *bios*, life; N.L. neut. n. *Terribium*, a bacterium isolated from soil).

Cells are Gram-stain-negative, white-pigmented, aerobic, rod-shaped bacterium. The genus represents a distinct branch in the family *Phyllobacteriaceae* of the class *Alphaproteobacteria* based on the core-genomic ML phylogeny. The genome size of the type strain is 6.02 Mb and the DNA G + C content is 63.17%. The type species is *Terribium terrae*.

**Description of Terribium terrae comb. nov.**

*Terribium* terrae (ter′rae. L. gen. n. terrae indicating soil as the source of isolation).

Basonym: *Mesorhizobium terrae* Jung et al*.* 2021.

The description is the same as *M. terrae* [14]. The type strain is NIBRBAC000500504^T^ (= KCTC72278^T^ = JCM33432^T^) isolated from soil in Jangsu in Jeollabukdo, Korea. The DNA genome size is 6.02 Mb, the G + C content of the type strain is 63.17% (by genome).

4. Description of Allomesorhizobium gen. nov.

*Allomesorhizobium* (Al.lo.me.so.rhi.zo′bi.um. Gr.masc. *allos*, other; N.L. neut. n. *Mesorhizobium*, a bacterial genus name. N.L. neut. n. *Allomesorhizobium*, a new group phylogenetically separated from the genus *Mesorhizobium*).

Cells are Gram-staining-negative, aerobic, motile, rod-shaped bacterium. The genus represents a distinct branch in the family *Phyllobacteriaceae* of the class *Alphaproteobacteria* based on the core-genomic ML phylogeny. The genome size of the type strains is 6.97–7.30 M and the DNA G + C content is 62.41–62.65%. The type species is *Allomesorhizobium alhagi*.

**Description of Allomesorhizobium alhagi comb. nov.**

*Allomesorhizobium alhagi* (al.ha'gi. N.L. gen. n. *alhagi* of *Alhagi,* a genus of leguminous plants, referring to the host from which the type strain was isolated).

Basonym: *Mesorhizobium alhagi* Chen et al*.* 2010.

The description is the same as *M. alhagi* [68]. The type strain, CCNWXJ12-2^ T^ (= ACCC 15461^ T^ = HAMBI 3019^ T^), was isolated from a root nodule of *Alhagi sparsifolia* in Alaer, Xinjiang, China. The genome size of the type strain is 6.97 Mb and the DNA G + C content is 62.65% (by genome).

**Description of Allomesorhizobium camelthorni comb. nov.**

*Allomesorhizobium camelthorni* (ca.mel.thor′ni. N.L. neut. n. *camelthornum* camelthorn, a common name for leguminous plants of the genus *Alhagi* in China; N.L. gen. n. *camelthorni* of camelthorn, from which the type strain was isolated).

Basonym: *Mesorhizobium camelthorni* Chen et al*.* 2011.

The description is the same as *M. camelthorni* [3]. The type strain is CCNWXJ 40-4^ T^ (= HAMBI 3020^ T^ = ACCC 14549^ T^), was isolated from a root nodule of *Alhagi sparsifolia* in Alaer, Xinjiang Province, China. The genome size of the type strain is 7.30 Mb and the DNA G + C content is 62.41% (by genome).

5. Description of Kumtagia gen. nov.

*Kumtagia* (Kum.ta′gia. N.L. fem. n. *Kumtagia*, pertaining to the Kumtag Desert in northwest China, where the type strain was isolated).

Cells are Gram-stain-negative, non-spore-forming, facultative, rod-shaped bacterium. The genus represents a distinct branch in the family *Phyllobacteriaceae* of the class *Alphaproteobacteria* based on the core-genomic ML phylogeny. The genome size of the type strain is 6.11 Mb and the DNA G + C content is 66.43%. The type species is *Kumtagia ephedrae*.

**Description of Kumtagia ephedrae comb. nov.**

*Kumtagia ephedrae* (eph.e′drae. N.L. gen. n. *ephedrae* of *Ephedra*, referring to the generic name of *Ephedra przewalskii* from which the strain was isolated).

Basonym: *Mesorhizobium ephedrae* Liu et al*.* 2018.

The description is the same as *M. ephedrae* [17]. The type strain 6GN-30^ T^ (= ACCC 60073^ T^ = KCTC 62410^ T^) was isolated from root of *E. przewalskii* in Kumtag Desert, Xinjiang, PR China. The genome size of the type strain is 6.11 M and the DNA G + C content 66.43% (by genome).

6. Description of Borborobacter gen. nov.

*Borborobacter* (Bor.bo.ro.bac.ter. Gr. masc. n. borboros, mud, dirt; N.L. masc. n. *bacter*, rod; N.L masc. n. Borborobacter, a rod-shaped bacterium isolated from mud).

Cells are Gram-stain-negative, rod-shaped bacterium. The genus represents a distinct branch in the family *Phyllobacteriaceae* of the class *Alphaproteobacteria* based on the core-genomic ML phylogeny. The genome size of the type strain is 5.21 Mb and the DNA G + C content is 61.42%. The type species is *Pseudaminobacter arsenicus*.

**Description of Borborobacter arsenicus comb. nov.**

*Borborobacter arsenicus* (ar.se′ni.cus. N.L. masc. adj. arsenicus, pertaining to arsenic).

Basonym: *Pseudaminobacter arsenicus* Mu et al. 2019.

The description is the same as *P. arsenicus* [29]. The type strain is CB3T (= CCTCC AB2016116T = KCTC 52625 T) isolated from arsenic-rich aquifers at the Jianghan Plain in Hubei, China. The genome size is 5.21 Mb and the DNA G + C content is 61.42%.

7. Description of Neomesorhizobium gen. nov.

*Neomesorhizobium* (Ne.o.me.so.rhi.zo′bi.um. Gr. masc. adj. neos, new; N.L. neut. n. *Mesorhizobium*, a bacterial genus name. N.L. neut. n. *Neomesorhizobium*, a new genus separated from the genus *Mesorhizobium*).

Cells are Gram-negative, aerobic, motile, non-spore-forming rods. The genus represents a distinct branch in the family *Phyllobacteriaceae* of the class *Alphaproteobacteria* based on the core-genomic ML phylogeny. The genome size of the type strain is 6.27 Mb and the DNA G + C content is 62.08%. The type species is *Neomesorhizobium albiziae*.

**Description of Neomesorhizobium albiziae comb. nov**

*Neomesorhizobium albiziae* (al.bi'zi.ae. N.L. gen. fem. n. *albiziae* of *Albizia*, a genus of leguminous plants, referring to the isolation of the first strains from *Albizia kalkora*).

Basonym: *Mesorhizobium albiziae* Wang et al*.* 2007.

The description is the same as *M. albiziae* Wang et al. 2007 [67]. The type strain is CCBAU 61158^ T^ (= LMG 23507^ T^ = USDA 4964^ T^), and was isolated from root nodules of *Albizia kalkora*. The genome size is 6.27 M and the DNA G + C content is 62.08% (by genome).

8. Description of Neoaquamicrobium gen. nov.

*Neoaquamicrobium* (Ne.o.a.qua.mi.cro′bi.um. Gr. masc. adj. neos, new; N.L. neut. n. *Aquamicrobium*, a bacterial genus name. N.L. neut. n. *Neoaquamicrobium*, a new genus which bacteria also living in water).

Cells are Gram-negative, aerobic, motile, non-spore-forming rods. The genus represents a distinct branch in the family *Phyllobacteriaceae* of the class *Alphaproteobacteria* based on the core-genomic ML phylogeny. The genome size of the type strains is 4.84–6.14 Mb and the DNA G + C content is 63.27–64.14%. The type species is *Neoaquamicrobium sediminum*.

**Description of Neoaquamicrobium sediminum comb. nov.**

*Neoaquamicrobium sediminum* (se.di.mi′num. L. gen. pl. n. *sediminum* of sediments, pertaining to source of the isolate).

Basonym: *Mesorhizobium sediminum* Yuan et al*.* 2016.

The description is the same as *M. sediminum* [18]. The type strain is YIM M12096^T^ (= CCTCC AB 2014219^ T^ = KCTC 42205^ T^), isolated from deep-sea sediment collected from the Indian Ocean. The genome size is 6.14 M and the DNA G + C content of the type strain is 63.27% (by genome).

**Description of Neoaquamicrobium microcysteis comb. nov.**

*Neoaquamicrobium microcysteis* (mi.cro.cys'te.is. N.L. gen. n. *microcysteis*, of the cyanobacterial genus *Microcystis*).

Basonym: *Mesorhizobium microcysteis* Jung et al*.* 2021.

The description is the same as *M. microcysteis* [19]. The type strain is MaA-C15^T^ (= KACC 21226^ T^ = JCM 33503^ T^), isolated from a xenic culture of *Microcystis aeruginosa* in the Republic of Korea. The genome size is 4.84 M and the DNA G + C content of the type strain is 64.14% (by genome).

9. Description of Aerobium gen. nov.

*Aerobium* (Ae.ro'bi.um, Gr. masc. n. *aér*, air; Gr. masc. n. *bios*, life; N.L. neut. n. *Aerobium*, a bacterium isolated from air).

Cells are Gram-negative, aerobic, motile, rod-shaped. The genus represents a distinct branch in the family *Phyllobacteriaceae* of the class *Alphaproteobacteria* based on the core-genomic ML phylogeny. The genome size of the type strains is 3.64 Mb and the DNA G + C content is 60.6%. The type species is *Aerobium aerolatum*.

**Description of Aerobium aerolatum comb. nov.**

*Aerobium aerolatum* (ae.ro.la′tum. Gr. Masc. n. *aer* air; L. part. adj. *latus* -*a* -*um* carried; N.L. neut. part. adj. *aerolatum* airborne).

Basonym: *Aquamicrobium aerolatum* Kämpfer et al*.* 2009.

The description is the same as *Aquamicrobium aerolatum* [22]. The type strain is DSM 21857^ T^ (= CCUG 57044^ T^ = Sa14^T^), isolated from air sampled in a duck shed. The genome size is 3.64 Mb and the DNA G + C content of the type strain is 60.06% (by genome).

10. Emended description of genus Mesorhizobium Jarvis et al. 1997

The description is as given by Jarvis et al. 1997 [2], with the following modifications: the genome sizes of the type strains are 6.20–8.58 Mb, and the genome G + C content varied from 61.84 to 64.00%.

**Emended description of Mesorhizobium sophorae **[10].

*Mesorhizobium sophorae* (so.pho′rae. N.L. fem. n. *Sophora*, botanical name of a genus of leguminous plants; N.L. gen. n. *sophorae*, of *Sophora*, referring to the host from which the type strain was isolated).

Basonym: *Mesorhizobium sophorae* De Meyer et al*.* 2016.

The description is as before [10] with the following addition. The genome size of the type strain is 8.49 Mb, and the DNA G + C content is 62.22% (by genome).

11. Emended description of genus Pseudaminobacter Kämpfer et al. 1999

*Pseudaminobacter* (Pseud.ami.no.bac.ter. Gr. adj. pseudos false; N.L. *Aminobacter*, generic name of a bacterium, N.L. masc. n. *Pseudaminobacter*, false Aminobacter).

The description is as given by Kämpfer et al. [28] with the following amendment: the genomic size is 4.84–6.27 Mb, the G + C content is 61.42–62.68% (by genome). The type species is *Pseudaminobacter salicylatoxidans*.

**Description of Pseudaminobacter soli comb. nov.**

*Pseudaminobacter soli* (so′li. L. gen. neut. n. soli, of the soil, the source of the type strain).

Basonym: *Mesorhizobium soli* Nguyen et al*.* 2015.

The description is the same as *M. soli* Nguyen et al. 2015 [15]. The type strain, NHI-8^ T^ (= KEMB 9005-153^ T^ = KACC 17916^ T^ = JCM 19897^ T^), was isolated from rhizosphere of legume tree *Robinia pseudoacacia* L. at Kyonggi University in Suwon, South Korea. The genomic size is 6.27 Mb, the G + C content is 62.57% (by genome).

12. Emended description of genus Corticibacterium Li et al. 2016

*Corticibacterium* (Cor.ti.ci.bac.te′ri.um. L. n. *cortex* bark; L. neut. n. *bacterium*, a rod; N. L. masc. n. *Corticibacterium* a rod from bark).

The description is as given by Li et al. [38] with the following emendations: the genome size is 3.83–4.29 Mb, the G + C content is 61.30–6.39% (by genome). The type species is *Corticibacterium populi*.

**Emended description of Corticibacterium populi Li et al. 2016**

*Corticibacterium populi* (po′pu.li. L. fem. gen. n. *populi* of *Populus*, the poplar tree).

Homotypic synonym: *Tianweitania populi* (Li et al., 2016) Song et al. 2023.

The description is the same as *Corticibacterium populi* Li et al. 2016 [38] and *Tianweitania populi* Song et al. 2023. [39]. The type strain is 16B10-2-7^ T^ (= CFCC 12884^ T^ = KCTC 42249^ T^), isolated from bark tissue of *Populus* × *euramericana*.

**Emended description of Corticibacterium aestuarii comb. nov**

*Corticibacterium aestuarii* (a. es. tu.a’ri.i. L. gen. n. *aestuarii* of the tidal flat, from where the type strain was isolated).

Basonym: *Tianweitania aestuarii* Song et al. 2023.

The description is the same as given by Song et al. 2023 [39]. The type strain is BSSL-BM11^T^ (= KACC 21634^ T^ = NBRC 114503^ T^), isolated from sand of a coastal dune at Boryeong on the Yellow Sea, Republic of Korea.

### Supplementary Information

**Supplementary Material 1.**

## Data Availability

All the genome sequences used in this study were downloaded from GenBank (https://ftp.ncbi.nlm.nih.gov/genomes/genbank/bacteria/) or GCM (https://gctype.wdcm.org/), and the deposit numbers are listed in Table S1.

## Abbreviations

ANI: Average nucleotide identity
AAI: Average amino acid identity
cAAI: Core-proteome average amino acid identity
GBDP: Genome BLAST distance phylogeny
GTDB: Genome taxonomy database
ML: Maximum likelihood
OGRIs: Overall genome relatedness indices
