# Supplementary material for: Phylogenomic analyses and reclassification of the Mesorhizobium complex: proposal for 9 novel genera and reclassification of 15 species
Source: BMC Genomics. 2024 Apr 29;25:419. doi: 10.1186/s12864-024-10333-y (PMC11057113; doi:10.1186/s12864-024-10333-y)
Supplement: Supplementary file 1 — Supplementary Material 1. [file 12864_2024_10333_MOESM1_ESM.pdf]

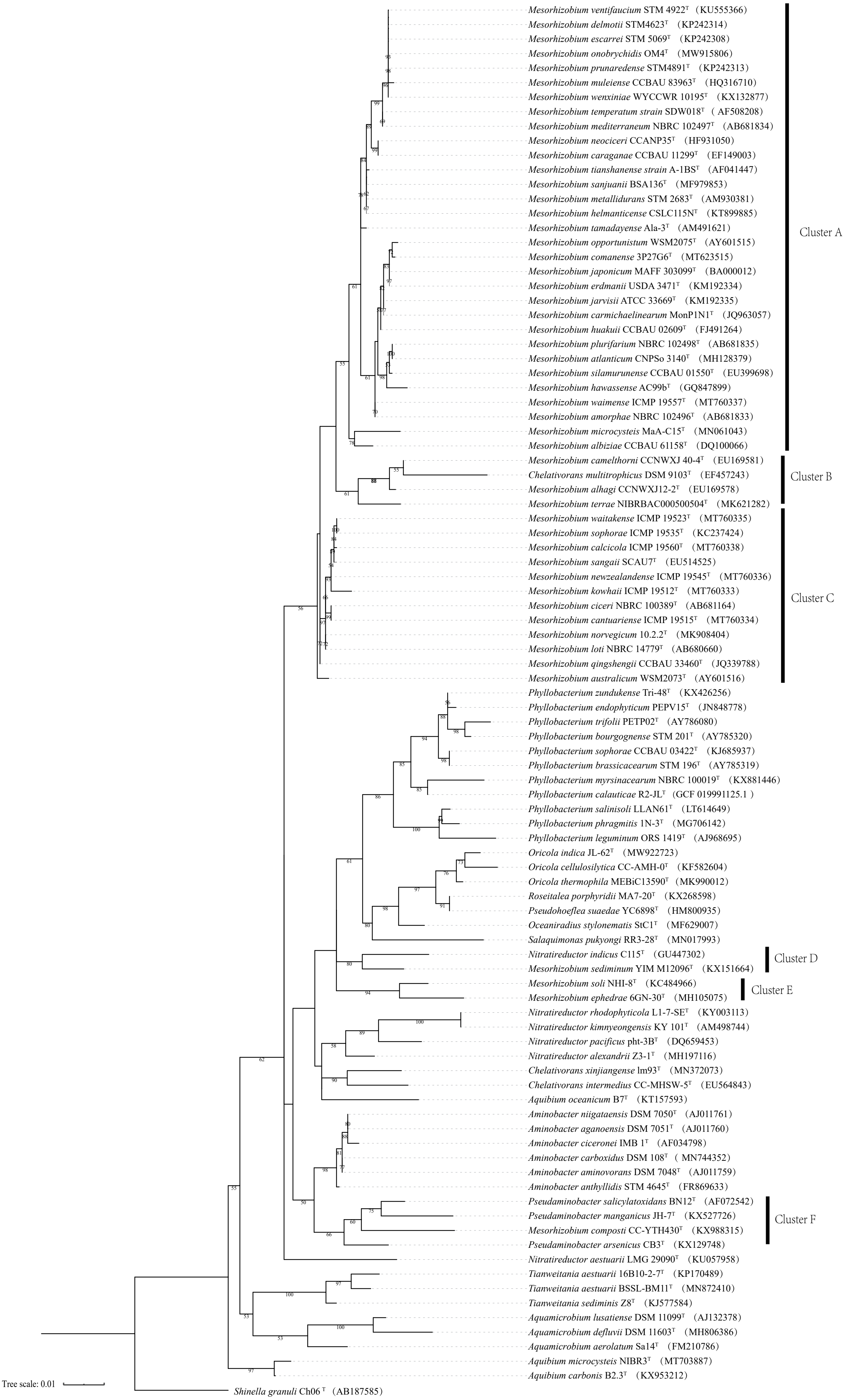

Fig. S1. Maximum likelihood (ML) phylogenomic tree based on the 16S rRNA gene sequences of *Phyllobacteriaceae* type strains. The tree was reconstructed using IQREE 2.0.3 with the best model TVM+F+I+R3, strain *Shinella granuli* DSM 18401^{T} was selected as an outgroup. Bootstrap values greaterthan 50% are shown at the nodes. Bar, 0.01 means 1% nucleotide substitution.

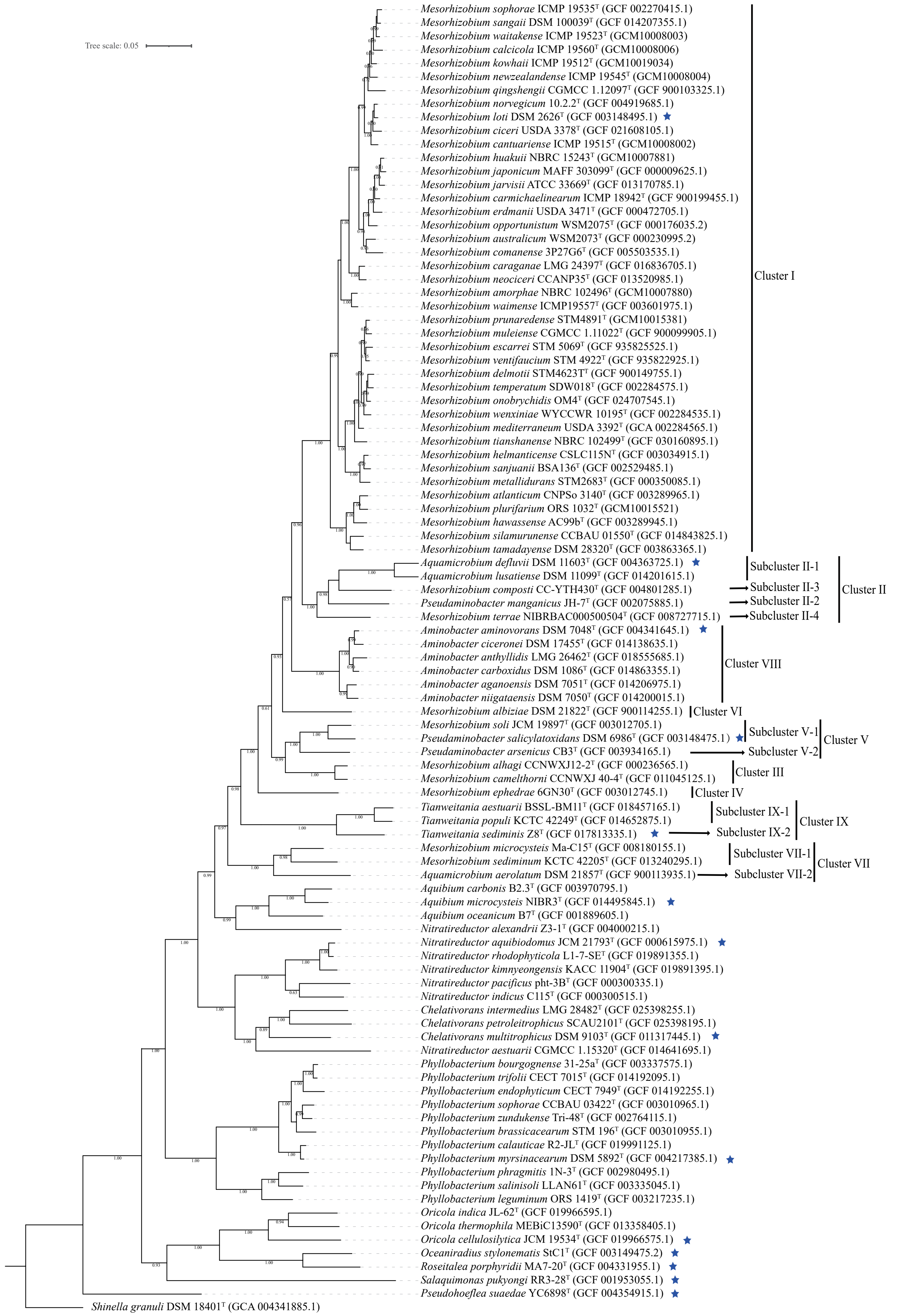

Fig. S2 Phylogenetic relationships between members of the *Phyllobacteriaceae* type strains by the GTDB. Type strains belong to the *Mesorhizobium* complex were reclassified and marked on the tree. The maximum likelihood (ML) tree was inferred based on alignment of 120 concatenated marker genes downloaded from GTDB R06-R202. Strains with blue asterisk, indicating type species of the genus.

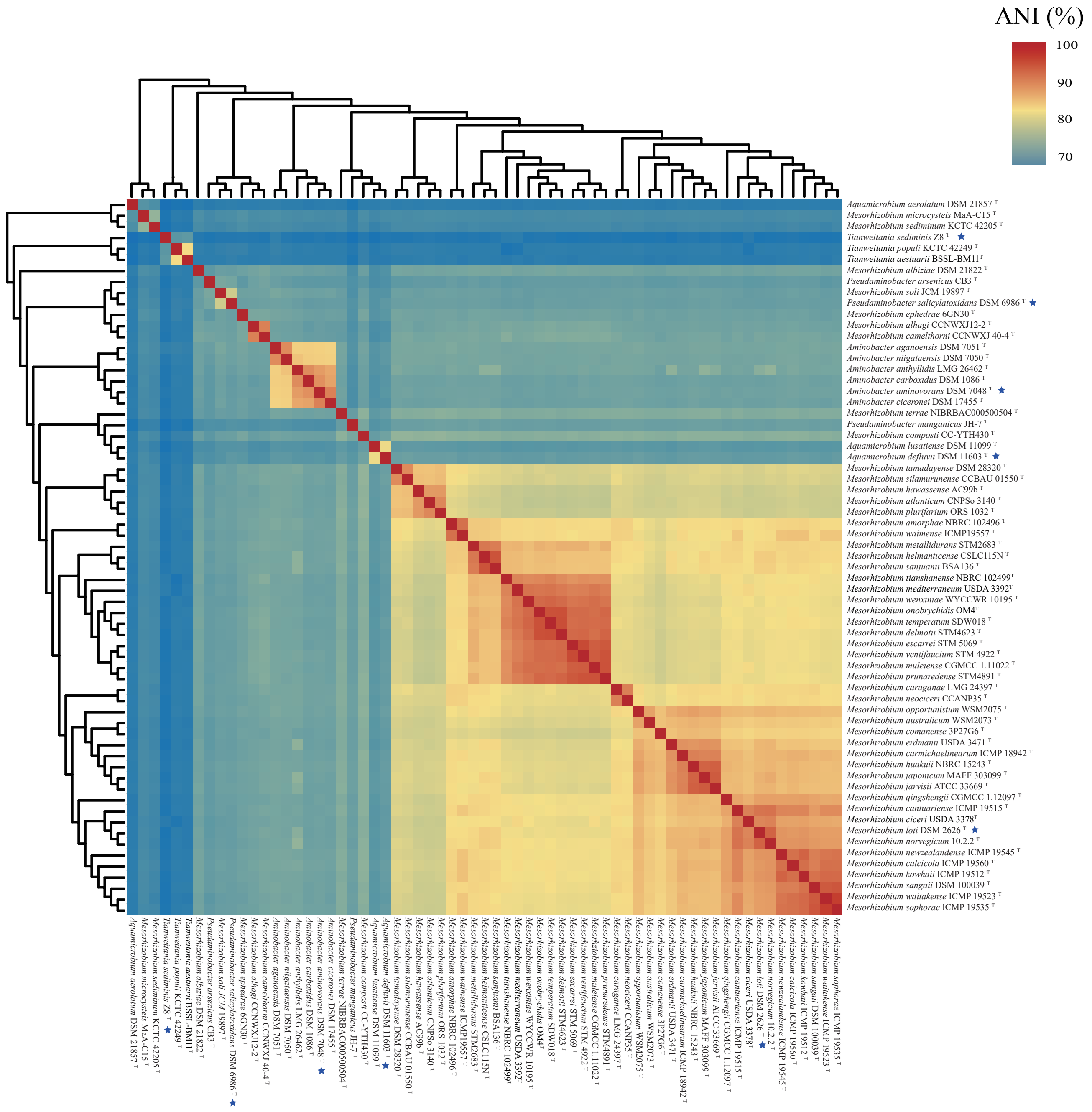

Fig. S3. Heatmap depicting the ANI values generated from all the genome pairs of the type strains belong to the *Mesorhizobium* complex. Type strains with blue asterisk marks represent type species.

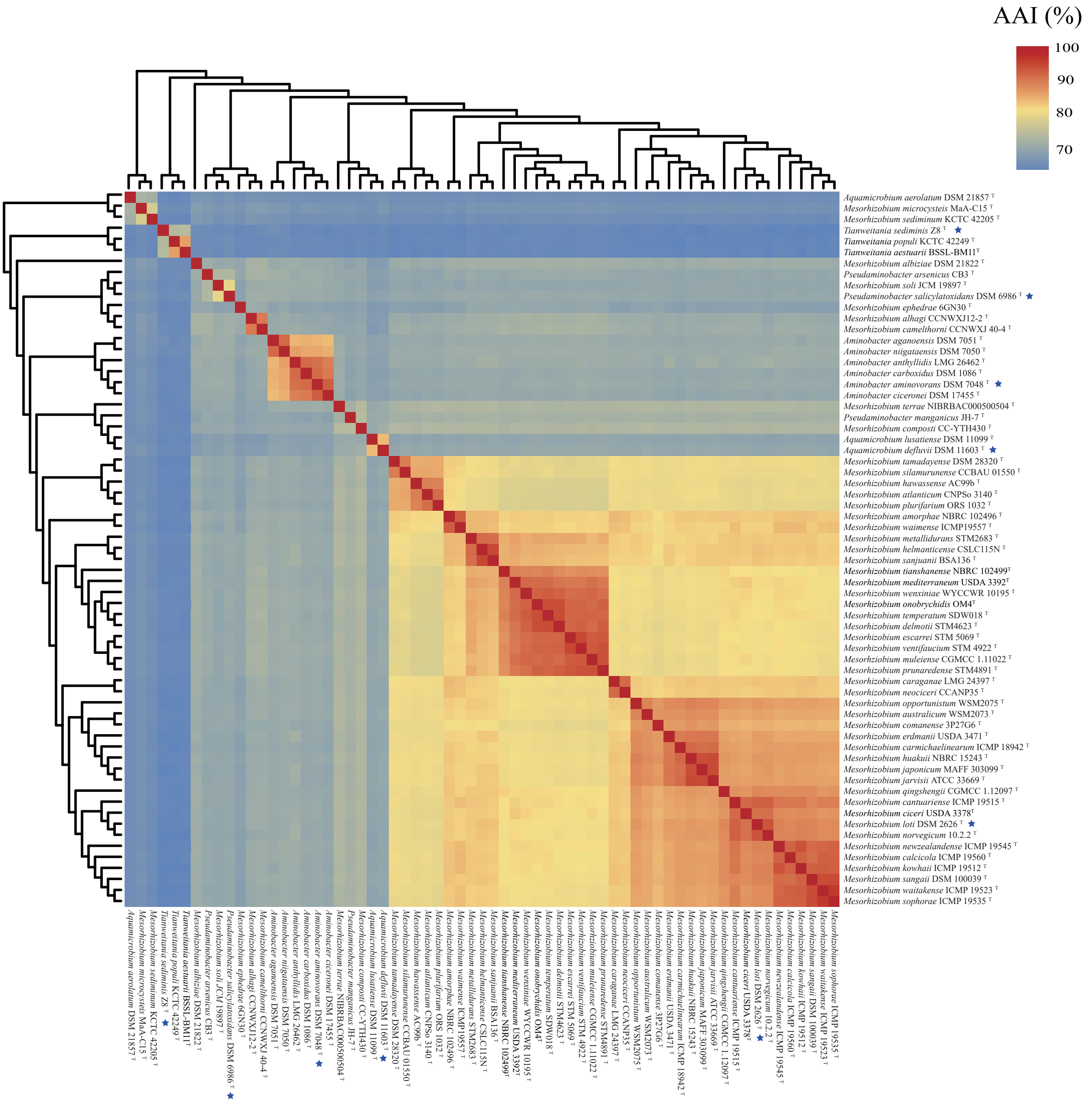

Fig. S4. Heatmap depicting the AAI values generated from all the genome pairs of the type strains belong to the *Mesorhizobium* complex. Type strains with blue asterisk marks represent type species.

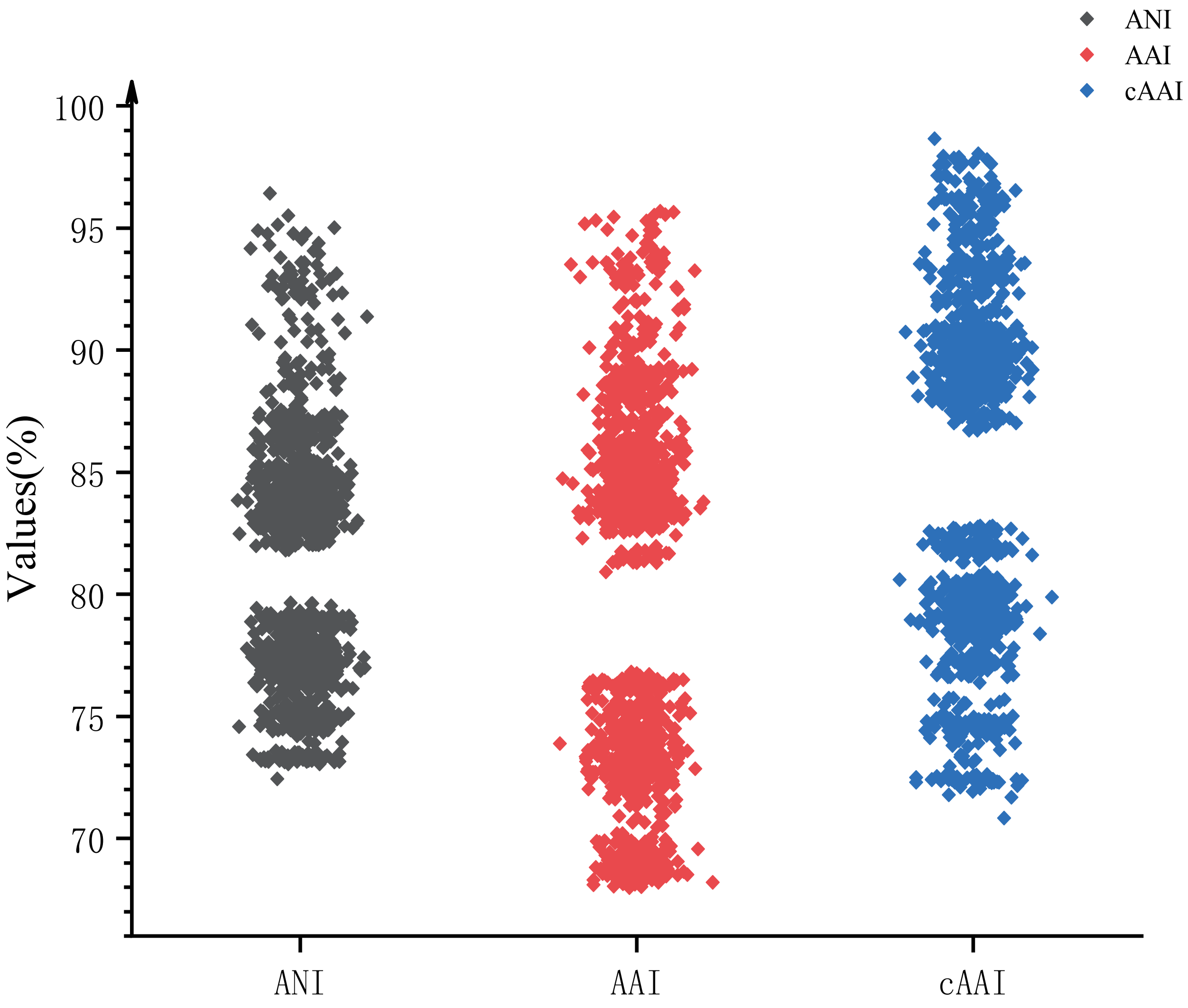

Fig. S5. The ANI, AAI and cAAI values generated between all the genome pairs of the type strains belong to *Mesorhizobium* complex.

Table S1 Genome information of *Phyllobacteriaceae* type strains used in this study.

Strains	Accession number	Size (Mb)	Contig number	Genome type	G+C (%)	N50 (bp)	Completeness	Contamination
Mesorhizobium sophorae ICMP 19535^{T}	GCF 002270415.1	8.05	24	draft	62.22	666711	98.68	3.19
Mesorhizobium waitakense ICMP 19523^{T}	GCM10008003$^{\Delta}$	8.17	244	draft	62.09	92945	99.51	2.29
Mesorhizobium sangaii DSM 100039^{T}	GCF 014207355.1	7.21	39	draft	62.38	1021540	99.51	0.41
Mesorhizobium kowhaii ICMP 19512^{T}	GCM10019034$^{\Delta}$	8.45	220	draft	62.12	272000	99.51	1.13
Mesorhizobium calcicola ICMP 19560^{T}	GCM10008006$^{\Delta}$	7.61	207	draft	62.54	186063	99.51	1.47
Mesorhizobium newzealandense ICMP 19545^{T}	GCM10008004$^{\Delta}$	7.66	170	draft	62.37	279087	99.51	1.06
Mesorhizobium norvegicum 10.2.2^{T}	GCF 004919685.1	8.27	249	draft	62.41	103508	98.68	0.99
Mesorhizobium loti DSM 2626$^{T\bullet}$	GCF 003148495.1	7.45	52	draft	62.35	607128	99.51	0.72
Mesorhizobium ciceri USDA 3378^{T}	GCF 021608105.1	6.91	96	draft	62.55	280282	99.09	1.19
Mesorhizobium cantuariense ICMP 19515^{T}	GCM10008002$^{\Delta}$	7.60	242	draft	62.35	119502	99.51	0.65
Mesorhizobium qingshengii CGMCC 1.12097^{T}	GCF 900103325.1	7.06	121	draft	62.66	254545	99.92	0.72
Mesorhizobium jarvisii ATCC 33669^{T}	GCF 013170785.1	7.20	3	complete	62.91	--	99.92	0.14
Mesorhizobium japonicum MAFF 303099^{T}	GCF 000009625.1	7.60	3	complete	62.51	--	99.92	2.05
Mesorhizobium huakuii NBRC 15243^{T}	GCM10007881$^{\Delta}$	6.84	90	draft	63.20	219594	99.51	0.68
Mesorhizobium carmichaelinearum ICMP 18942^{T}	GCF 900199455.1	8.58	233	draft	62.39	328797	99.92	1.57
Mesorhizobium erdmanii USDA 3471^{T}	GCF 000472705.1	7.02	50	draft	62.66	316419	99.51	0.16
Mesorhizobium comanense 3P27G6^{T}	GCF 005503535.1	7.36	76	draft	63.53	262341	99.51	2.12
Mesorhizobium australicum WSM2073^{T}	GCF 000230995.2	6.20	1	complete	62.84	--	99.51	0.39
Mesorhizobium opportunistum WSM2075^{T}	GCF 000176035.2	6.88	1	complete	62.87	--	99.92	1.20
Mesorhizobium neociceri CCANP35^{T}	GCF 013520985.1	7.29	72	draft	62.37	294534	99.51	1.48
Mesorhizobium caraganae LMG 24397^{T}	GCF 016836705.1	7.30	170	draft	62.33	177546	99.51	0.89
Mesorhizobium prunaredense STM4891^{T}	GCM10015381$^{\Delta}$	7.01	82	draft	62.06	321527	100	2.94

Mesorhziobium muleiense CGMCC 1.11022^{T}	GCF 900099905.1	6.81	61	draft	62.28	257857	99.59	1.98
Mesorhizobium ventifaucium STM 4922^{T}	GCF 935822925.1	6.74	96	draft	62.10	152300	99.55	1.57
Mesorhizobium escarrei STM 5069^{T}	GCF 935825525.1	7.71	200	draft	62.00	93056	99.51	1.88
Mesorhizobium delmotii STM4623^{T}	GCF 900149755.1	7.60	113	draft	61.85	234211	99.59	3.63
Mesorhizobium temperatum SDW018^{T}	GCF 002284575.1	7.17	77	draft	61.90	254929	99.59	1.61
Mesorhizobium onobrychidis OM4^{T}	GCF 024707545.1	7.55	2	complete	61.88	--	99.59	1.84
Mesorhizobium wenxiniae WYCCWR 10195^{T}	GCF 002284535.1	6.68	43	draft	61.88	386068	99.18	1.64
Mesorhizobium mediterraneum USDA 3392 ^{T}	GCA 002284565.1	7.20	52	draft	62.01	269395	99.59	0.82
Mesorhizobium tianshanense NBRC 102499 ^{T}	GCF 030160895.1	7.86	159	draft	62.12	114779	99.55	1.24
Mesorhizobium sanjuanii BSA136^{T}	GCF 002529485.1	6.40	343	draft	62.51	35232	99.51	1.31
Mesorhizobium helmanticense CSLC115N^{T}	GCF 003034915.1	6.88	145	draft	62.43	181688	99.51	1.40
Mesorhizobium metallidurans STM2683^{T}	GCF 000350085.1	6.23	191	draft	62.45	62019	98.69	1.71
Mesorhizobium waimense ICMP19557^{T}	GCF 003601975.1	8.15	290	draft	62.39	169793	99.71	1.76
Mesorhizobium amorphae NBRC 102496^{T}	GCM10007880$^{\Delta}$	7.20	106	draft	62.87	477862	99.92	1.07
Mesorhizobium plurifarium ORS 1032^{T}	GCM10015521$^{\Delta}$	7.01	52	draft	64.00	306043	98.41	0.19
Mesorhizobium atlanticum CNPSo 3140^{T}	GCF 003289965.1	6.54	29	draft	63.73	776796	99.51	2.70
Mesorhizobium hawassense AC99b^{T}	GCF 003289945.1	7.34	71	draft	63.35	303349	99.10	0.61
Mesorhizobium silamurunense CCBAU 01550^{T}	GCF 014843825.1	7.04	493	draft	63.10	27072	99.02	0.90
Mesorhizobium tamadayense DSM 28320^{T}	GCF 003863365.1	7.53	303	draft	63.17	99038	98.69	0.61
Aquamicrobium defluvii DSM 11603$^{T\bullet}$	GCF 004363725.1	4.52	91	draft	63.15	132816	99.51	1.24
Aquamicrobium lusatiense DSM 11099^{T}	GCF 014201615.1	4.39	13	draft	62.60	2914411	98.89	1.16
Mesorhizobium composti CC-YTH430^{T}	GCF 004801285.1	4.65	36	draft	65.18	314054	99.3	0.89
Pseudaminobacter manganicus JH-7^{T}	GCF 002075885.1	4.84	60	draft	61.20	152861	99.18	0.71
Mesorhizobium terrae NIBRBAC000500504^{T}	GCF 008727715.1	6.02	2	complete	63.17	--	99.51	0.82
Aminobacter ciceronei DSM 17455^{T}	GCF 014138635.1	6.78	96	draft	63.07	156335	99.51	0.82
Aminobacter aminovorans DSM 7048$^{T\bullet}$	GCF 004341645.1	5.85	29	draft	63.20	467483	99.30	1.13

Aminobacter carboxidus DSM 1086^{T}	GCF 014863355.1	6.29	31	draft	62.96	458931	99.30	0.30
Aminobacter anthyllidis LMG 26462^{T}	GCF 018555685.1	6.72	30	draft	62.58	670596	99.30	0.55
Aminobacter niigataensis DSM 7050^{T}	GCF 014200015.1	5.29	26	draft	63.40	468746	99.30	2.40
Aminobacter aganoensis DSM 7051^{T}	GCF 014206975.1	5.77	52	draft	63.89	248423	99.51	0.55
Pseudaminobacter salicylatoxidans DSM 6986^{T} •	GCF 003148475.1	4.84	43	draft	62.68	232367	99.02	0.61
Mesorhizobium soli JCM 19897^{T}	GCF 003012705.1	6.27	118	draft	62.57	336579	99.59	1.02
Pseudaminobacter arsenicus CB3^{T}	GCF 003934165.1	5.21	107	draft	61.42	241998	99.30	0.31
Mesorhizobium camelthorni CCNWXJ 40-4^{T}	GCF 011045125.1	7.30	346	draft	62.41	74213	99.51	0.44
Mesorhizobium alhagi CCNWXJ12-2^{T}	GCF 000236565.1	6.97	375	draft	62.65	35501	99.02	2.25
Mesorhizobium ephedrae 6GN30^{T}	GCF 003012745.1	6.11	115	draft	66.43	165777	99.02	2.13
Mesorhizobium albiziae DSM 21822^{T}	GCF 900114255.1	6.27	92	draft	62.08	246691	99.51	1.64
Tianweitania aestuarii BSSL-BM11 ^{T}	GCF 018457165.1	3.83	7	draft	61.30	801338	99.18	0.82
Tianweitania sediminis Z8^{T} •	GCF 017813335.1	4.70	22	draft	61.83	474629	99.71	2.15
Tianweitania populi KCTC 42249^{T} •	GCF 014652875.1	4.29	16	draft	61.39	1463426	99.59	1.40
Aquamicrobium aerolatum DSM 21857^{T}	GCF 900113935.1	3.64	49	draft	60.06	170747	99.18	0.63
Mesorhizobium sediminum KCTC 42205^{T}	GCF 013240295.1	6.14	9	draft	63.27	2224989	98.69	1.13
Mesorhizobium microcysteis MaA-C15^{T}	GCF 008180155.1	4.84	68	draft	64.14	287578	96.30	3.09
Nitratireductor alexandrii Z3-1^{T}	GCF 004000215.1	4.87	40	draft	65.61	547451	99.59	1.15
Aquibium carbonis B2.3^{T}	GCF 003970795.1	5.35	188	draft	66.05	60530	99.59	1.64
Aquibium microcysteis NIBR3^{T} ●	GCF 014495845.1	6.11	1	complete	67.90	--	99.43	2.20
Aquibium oceanicum B7^{T}	GCF 001889605.1	5.32	2	complete	65.06	--	99.43	1.38
Nitratireductor pacificus pht-3B^{T}	GCF 000300335.1	4.47	51	draft	65.51	151309	98.68	0.08
Nitratireductor indicus C115^{T}	GCF 000300515.1	4.99	75	draft	60.85	260779	99.51	0.18
Nitratireductor aquibiodomus JCM 21793^{T} •	GCF 000615975.1	4.52	207	draft	61.35	40247	99.10	0.46
Nitratireductor rhodophyticola L1-7-SE^{T}	GCF 019891355.1	4.40	18	draft	61.66	1135733	98.77	1.11
Nitratireductor kimnyeongensis KACC 11904^{T}	GCF 019891395.1	4.05	2	complete	59.80	--	99.51	1.97

Chelativorans intermedius LMG 28482^T	GCF 025398255.1	4.19	103	draft	65.70	159574	99.18	2.01
Chelativorans multitrophicus DSM 9103^T •	GCF 011317445.1	4.82	15	draft	61.03	778588	99.18	0.24
Chelativorans petroleitrophicus SCAU2101^T	GCF 025398195.1	3.97	70	draft	63.08	187496	99.18	0.23
Nitratireductor aestuarii CGMCC 1.15320^T	GCF 014641695.1	4.52	74	draft	60.28	294970	99.18	2.83
Phyllobacterium zundukense Tri-48^T	GCF 002764115.1	6.21	6	complete	57.22	--	99.93	0.55
Phyllobacterium sophorae CCBAU 03422^T	GCF 003010965.1	6.36	49	draft	57.04	289216	99.57	0.43
Phyllobacterium brassicacearum STM 196^T	GCF 003010955.1	5.79	87	draft	57.32	192543	99.57	0.87
Phyllobacterium bourgognense 31-25a^T	GCF 003337575.1	5.62	61	draft	56.44	228269	100	0
Phyllobacterium trifolii CECT 7015^T	GCF 014192095.1	6.31	69	draft	56.4	236202	99.57	0
Phyllobacterium endophyticum CECT 7949^T	GCF 014192255.1	5.51	9	draft	57.00	1246362	99.57	0
Phyllobacterium calauticae R2-JL^T	GCF 019991125.1	5.29	45	draft	59.11	720379	99.78	0
Phyllobacterium myrsinacearum DSM 5892^T •	GCF 004217385.1	5.42	17	draft	59.33	1327176	99.94	0.65
Phyllobacterium salinisoli LLAN61^T	GCF 003335045.1	5.06	188	draft	59.88	519422	99.94	0.43
Phyllobacterium phragmitis 1N-3^T	GCF 002980495.1	5.63	139	draft	60.11	200258	100	0.58
Phyllobacterium leguminum ORS 1419^T	GCF 003217235.1	3.74	68	draft	59.85	139063	99.78	0.43
Oricola indica JL-62^T	GCF 019966595.1	5.09	42	draft	62.09	422528	100	0
Oricola thermophila MEBiC13590^T	GCF 013358405.1	4.00	1	complete	63.54	--	99.61	1.10
Oricola cellulosilytica JCM 19534^T •	GCF 019966575.1	3.70	12	draft	61.47	513777	99.22	0
Oceaniradius stylonematis StC1^T •	GCF 003149475.2	3.58	23	draft	64.75	444929	99.38	0
Roseitalea porphyridii MA7-20^T •	GCF 004331955.1	3.54	1	complete	66.56	--	97.76	0.19
Salaquimonas pukyongi RR3-28^T •	GCF 001953055.1	3.36	1	complete	58.59	--	99.73	0.19
Pseudohoeflea suaedae YC6898^T •	GCF 004354915.1	3.77	21	draft	62.54	2102496	100	0.80

Note: Strain with "•", indicating it is the type species of the genus.

Table S2 Inter-cluster ANI, AAI and cAAI values between each cluster pairs.

(Sub) Cluster pairs	Inter-(sub)cluster ANI (%)	Inter-(sub)cluster ANI average (%)	Inter-(sub)cluster AAI (%)	Inter-(sub)cluster AAI average (%)	Inter-(sub)cluster cAAI (%)	Inter-(sub)cluster cAAI average (%)
Cluster I-Cluster II	75.98-79.64	77.61	72.18-76.82	74.72	78.86-83.98	80.79
Cluster I-Subcluster II-1	75.98-77.25	77.25	72.18-73.64	72.91	78.86-80.60	79.27
Cluster I- Subcluster II-2	78.74-79.64	79.08	75.87-76.82	76.37	81.66-83.41	82.07
Cluster I- Subcluster II-3	76.92-77.41	77.15	74.69-75.75	75.22	80.27-82.01	80.64
Cluster I- Subcluster II-4	78.35-79.10	78.70	75.63-76.57	76.21	82.23-83.98	82.70
Cluster I- Cluster III	77.30-78.88	77.92	73.24-75.97	74.37	79.71-81.35	80.20
Cluster I- Cluster IV	77.03-77.93	77.49	71.51-73.28	72.35	77.18-79.11	77.58
Cluster I- Cluster V	76.38-77.42	76.96	72.40-74.07	73.15	78.44-80.24	79.07
Cluster I- Subcluster V-1	76.78-77.42	77.08	72.40-73.76	73.10	78.44-80.24	79.03
Cluster I- Subcluster V-2	76.38-76.97	76.71	72.40-74.07	73.25	78.79-79.92	79.15
Cluster I- Cluster VI	77.31-78.09	77.75	73.50-75.03	74.43	79.22-80.88	79.60
Cluster I- Cluster VII	73.05-75.32	74.28	68.01-70.07	68.86	72.46-75.83	74.23
Cluster I- Subcluster VII-1	74-20-75.32	74.78	68.01-70.07	68.97	74.59-75.83	74.91
Cluster I- Subcluster VII-2	73.05-73.51	73.29	68.31-69.07	68.64	72.46-74.23	72.86
Cluster I- Cluster VIII	77.04-79.52	77.69	72.49-75.13	73.66	79.80-81.49	80.26
Cluster I- Cluster IX	72.26-73.69	73.10	66.70-67.87	67.27	72.09-73.54	72.46
Cluster I- Subcluster IX-1	72.30-73.69	73.34	66.74-67.87	67.29	72.09-73.45	72.47
Cluster I- Subcluster IX-2	72.26-73.36	72.61	66.70-67.70	67.21	72.17-73.54	72.43
Cluster II- Cluster III	75.18-77.18	76.06	70.66-73.08	72.03	76.81-78.70	77.68
Cluster II- Cluster IV	75.75-77.96	76.61	70.66-72.42	71.54	75.75-77.25	76.36
Cluster II- Cluster V	75.18-77.96	76.24	71.66-73.37	72.66	77.07-78.54	77.60
Cluster II- Subcluster V-1	75.18-77.18	76.06	70.66-73.08	72.03	77.10-78.54	77.60

Cluster II- Subcluster V-2	75.75-77.96	76.62	70.66-72.42	71.54	77.07-78.23	77.60
Cluster II- Cluster VI	75.24-77.19	76.07	70.86-73.23	72.18	76.57-78.62	77.40
Cluster II- Cluster VII	72.42-75.28	74.12	68.52-70.20	68.18	72.07-74.58	73.60
Cluster II- Subcluster VII-1	72.42-74.85	73.79	68.52-69.40	68.95	73.61-74.58	74.15
Cluster II- Subcluster VII-2	73.95-75.28	74.78	69.11-70.20	69.62	72.07-72.70	72.50
Cluster II- Cluster VIII	75.32-77.90	76.49	71.84-74.22	72.74	77.26-79.78	78.16
Cluster II- Cluster IX	72.52-73.85	72.97	67.18-67.97	67.58	71.46-72.28	71.92
Cluster II- Subcluster IX-1	72.52-73.86	73.23	67.31-67.97	67.61	71.46-72.28	71.90
Cluster II- Subcluster IX-2	71.94-72.77	72.45	67.18-67.72	67.53	71.64-72.24	71.94
Subcluster II-1- Subcluster II-2	76.87-77.47	77.17	73.61-74.37	73.99	79.45-79.64	79.55
Subcluster II-1- Subcluster II-3	75.97-76.21	76.09	73.71-73.97	73.84	78.61-78.95	78.78
Subcluster II-1- Subcluster II-4	76.52-76.83	76.68	73.35-73.79	73.57	79.01-79.14	79.08
Subcluster II-1- Cluster III	75.18-75.64	75.42	70.66-71.34	71.05	76.81-77.07	76.93
Subcluster II-1- Cluster IV	75.85-76.31	76.08	70.66-70.92	70.79	75.79-75.92	75.86
Subcluster II-1- Cluster V	75.22-76.49	75.84	71.54-72.49	71.94	77.07-77.23	77.14
Subcluster II-1- Subcluster V-1	75.89-76.49	76.07	71.54-72.49	71.95	77.10-77.17	77.13
Subcluster II-1- Subcluster V-2	75.22-75.52	75.37	71.72-72.10	71.91	77.07-77.23	77.15
Subcluster II-1- Cluster VI	75.25-75.72	75.49	70.86-71.48	71.17	76.57-76.70	76.64
Subcluster II-1- Cluster VII	73.32-75.08	74.16	68.66-70.20	69.28	72.51-74.26	73.53
Subcluster II-1- Subcluster VII-1	73.98-75.08	74.82	68.66-70.20	69.66	73.89-74.26	74.03
Subcluster II-1- Subcluster VII-2	73.32-73.59	73.46	69.20-69.40	69.3	72.51-72.59	72.55
Subcluster II-1- Cluster VIII	75.61-76.49	76.01	71.84-72.49	72.10	77.35-77.59	77.47
Subcluster II-1- Cluster IX	72.19-73.41	72.90	67.19-67.97	67.58	71.65-71.99	71.83
Subcluster II-1- Subcluster IX-1	72.96-73.41	73.15	67.31-67.97	67.64	71.65-71.99	71.86
Subcluster II-1- Subcluster IX-2	72.19-72.65	72.42	67.19-67.73	76.45	71.71-71.90	71.81
Subcluster II-2- Subcluster II-3	78.03	--	77.4	--	81.02	--

Subcluster II-2- Subcluster II-4	79.24	--	77.02	--	82.09	--
Subcluster II-2- Cluster III	76.93-77.17	77.06	72.68-73.08	72.88	78.46-78.54	78.50
Subcluster II-2- Cluster IV	77.96	--	72.42	--	77.25	--
Subcluster II-2- Cluster V	76.33-77.16	76.87	73.01-73.32	73.15	78.10-78.23	78.17
Subcluster II-2- Subcluster V-1	77.10-77.16	77.13	73.01-73.32	73.17	78.10-78.18	78.14
Subcluster II-2- Subcluster V-2	76.33	--	73.13	--	78.23	--
Subcluster II-2- Cluster VI	77.19	--	73.23	--	78.2	--
Subcluster II-2- Cluster VII	73.39-75.28	74.51	68.52.69.34	68.83	72.70-74.58	73.88
Subcluster II-2- Subcluster VII-1	74.85-75.28	75.06	68.52-69.34	68.93	74.35-74.58	74.47
Subcluster II-2- Subcluster VII-2	73.39	--	68.64	--	72.7	--
Subcluster II-2- Cluster VIII	77.08-77.73	77.32	72.84-73.33	73.02	78.55-78.81	78.65
Subcluster II-2- Cluster IX	72.77-73.85	73.49	67.62-67.92	67.75	72.04-72.28	72.18
Subcluster II-2- Subcluster IX-1	73.84-73.85	73.84	67.72-67.92	67.85	72.04-72.28	72.16
Subcluster II-2- Subcluster IX-2	72.77	--	67.62	--	72.23	--
Subcluster II-3- Subcluster II-4	76.91	--	75.09	--	80.11	--
Subcluster II-3- Cluster III	75.53-75.77	75.65	72.47-72.62	72.55	77.34-77.37	77.36
Subcluster II-3- Cluster IV	75.75	--	71.65	--	75.75	--
Subcluster II-3- Cluster V	75.55-75.90	75.7	72.64-73.31	73.04	77.13-77.36	77.27
Subcluster II-3- Subcluster V-1	75.55-75.90	75.72	72.64-73.18	72.91	77.13-77.32	77.23
Subcluster II-3- Subcluster V-2	75.66	--	73.31	--	77.36	--
Subcluster II-3- Cluster VI	75.32	--	72.13	--	76.90	--
Subcluster II-3- Cluster VII	72.43-73.95	73.43	68.74-69.82	69.27	72.07-73.78	73.15
Subcluster II-3- Subcluster VII-1	73.91-73.95	73.93	69.26-69.82	69.54	73.61-73.78	73.70
Subcluster II-3- Subcluster VII-2	72.43	--	68.74	--	72.07	--
Subcluster II-3- Cluster VIII	75.32-75.91	75.52	72.01-72.80	72.45	77.44-77.75	77.58
Subcluster II-3- Cluster IX	71.94-72.78	72.41	67.32-67.54	67.42	71.46-71.64	71.54

Subcluster II-3- Subcluster IX-1	72.52-72.78	72.64	67.32-67.40	67.36	71.46-71.52	71.49
Subcluster II-3- Subcluster IX-1	71.94	--	67.54	--	71.64	--
Subcluster II-4- Cluster III	76.72-76.77	76.75	72.57-72.68	72.62	78.63-78.70	78.67
Subcluster II-4- Cluster IV	77.2	--	72.06	--	77.08	--
Subcluster II-4- Cluster V	76.47-77.02	76.79	73.04-73.37	73.22	78.11-78.54	78.28
Subcluster II-4- Subcluster V-1	76.88-77.02	76.95	73.27-73.37	73.32	78.19-78.54	78.37
Subcluster II-4- Subcluster V-2	76.47	--	73.04	--	78.11	--
Subcluster II-4- Cluster VI	76.85	--	73.18	--	78.62	--
Subcluster II-4- Cluster VII	73.25-75.03	74.35	68.85-69.65	69.21	72.61-74.57	73.29
Subcluster II-4- Subcluster VII-1	74.76-75.03	74.89	69.14-69.65	69.4	74.48-74.57	74.53
Subcluster II-4- Subcluster VII-2	73.25	--	68.85	--	72.61	--
Subcluster II-4- Cluster VIII	77.35-7.90	77.57	73.83-74.22	74.03	79.48-79.78	79.65
Subcluster II-4- Cluster IX	72.71-73.39	73.14	67.58-67.61	67.69	72.07-72.24	72.18
Subcluster II-4- Subcluster IX-1	73.32-73.39	73.36	67.58-67.61	67.60	72.07-72.24	72.16
Subcluster II-4- Subcluster IX-2	72.71	--	67.58	--	72.24	--
Cluster III- Cluster IV	78.06-78.12	78.09	73.67-74.02	73.85	79.26-79.39	79.33
Cluster III- Cluster V	77.19-77.70	77.42	73.88-75.68	74.69	80.32-81.59	80.84
Cluster III- Subcluster V-1	77.34-77.48	77.36	73.88-74.67	74.28	80.32-80.65	80.48
Cluster III- Subcluster V-2	77.38-77.70	77.54	75.38-75.68	75.52	81.59	81.59
Cluster III- Cluster VI	77.65-78.04	77.85	75.31-75.51	75.41	80.07-80.09	80.08
Cluster III- Cluster VII	73.29-75.29	74.5	69.37-71.18	70.19	73.49-75.83	74.99
Cluster III- Subcluster VII-1	75.17-75.29	75.05	69.84-71.19	70.57	75.61-75.83	75.71
Cluster III- Subcluster VII-2	73.29-73.52	73.4	69.37-69.53	69.45	73.49-73.62	73.56
Cluster III- Cluster VIII	76.68-77.54	77.12	72.94-73.99	73.45	79.47-79.90	79.68
Cluster III- Cluster IX	72.63-73.34	73.08	67.75-68.10	67.88	72.93-73.13	73.01
Cluster III- Subcluster IX-1	73.21-73.34	73.30	67.75-67.97	67.82	73.00-73.13	73.04

Cluster III- Subcluster IX-2	72.63	72.63	67.91-68.10	68.01	72.93-72.97	72.95
Cluster IV- Cluster V	76.48-77.38	76.99	72.20-73.11	72.57	77.87-78.10	77.96
Cluster IV- Subcluster V-1	77.10-77.38	77.24	72.20-72.41	72.31	77.87-77.92	77.90
Cluster IV- Subcluster V-2	76.48	--	73.11	--	78.10	--
Cluster IV- Cluster VI	77.35	--	73.02	--	77.55	--
Cluster IV- Cluster VII	73.28-75.74	74.79	68.76-69.66	69.18	72.57-74.55	73.87
Cluster IV- Subcluster VII-1	75.35-75.74	75.55	69.11-69.66	69.39	74.48-74.55	74.52
Cluster IV- Subcluster VII-2	73.28	--	68.76	--	72.57	--
Cluster IV- Cluster VIII	77.12-77.82	77.42	71.98-72.95	72.29	77.05-77.43	77.19
Cluster IV- Cluster IX	72.80-73.91	73.34	67.40-67.77	67.61	72.07-72.21	72.16
Cluster IV- Subcluster IX-1	73.32-73.91	73.61	67.65-67.77	67.71	72.07-72.21	72.14
Cluster IV- Subcluster IX-2	72.80	--	67.40	--	72.20	--
Cluster V- Cluster VI	76.49-77.05	76.83	73.32-74.27	73.74	78.65-79.15	78.91
Cluster V- Cluster VII	73.14-75.33	74.6	69.07-70.68	69.85	73.32-75.54	74.60
Cluster V- Subcluster VII-1	74.46-75.33	74.84	69.04-70.68	70.01	75.02-75.45	75.20
Cluster V- Subcluster VII-2	73.14-73.52	73.35	69.07-69.82	69.53	73.32-73.47	73.41
Cluster V- Cluster VIII	76.40-7	77.06	73.51-74.68	74.00	78.95-79.39	79.13
Cluster V- Cluster IX	72.49-73.88	73.25	67.77-68.53	68.17	72.62-72.94	72.78
Cluster V- Subcluster IX-1	73.32-73.88	73.57	67.78-68.53	68.25	72.62-72.94	72.79
Cluster V- Subcluster IX-2	72.49-72.64	72.59	67.77-68.18	68.01	72.74-72.76	72.75
Subcluster V-1- Subcluster V-2	77.79-78.05	78.02	76.36-76.43	76.4	81.98-82.12	82.05
Subcluster V-1- Cluster VI	76.94-77.05	76.99	73.32-73.63	73.48	78.65-78.94	78.80
Subcluster V-1- Cluster VII	73.38-75.33	74.46	69.04-70.51	69.68	73.32-75.17	74.53
Subcluster V-1- Subcluster VII-1	74.46-75.33	74.96	69.04-70.51	69.84	75.02-75.17	75.11
Subcluster V-1- Subcluster VII-2	73.38-76.52	74.45	69.07-69.69	69.38	73.32-73.45	73.39
Subcluster V-1- Cluster VIII	76.69-78.10	77.29	73.51-74.26	73.95	78.95-79.39	79.16

Subcluster V-1- Cluster IX	72.64-73.88	73.33	67.77-68.53	68.14	72.67-72.91	72.78
Subcluster V-1- Subcluster IX-1	73.41-73.88	73.68	67.78-68.53	68.22	72.67-72.91	72.80
Subcluster V-1- Subcluster IX-2	72.64	72.64	67.77-68.18	67.98	72.74	72.74
Subcluster V-2- Cluster VI	76.49	--	74.27	--	79.15	--
Subcluster V-2- Cluster VII	73.14-74.63	74.11	69.82-70.68	70.17	73.47-75.45	74.74
Subcluster V-2- Subcluster VII-1	74.57-74.63	74.6	70.02-72.68	70.35	75.30-75.45	75.38
Subcluster V-2- Subcluster VII-2	73.14	--	69.82	--	73.47	--
Subcluster V-2- Cluster VIII	76.40-77.09	76.62	73.59-74.68	74.11	78.98-79.24	79.08
Subcluster V-2- Cluster IX	72.49-73.41	73.07	68.57-68.38	68.24	72.62-72.94	72.77
Subcluster V-2- Subcluster IX-1	73.32-73.41	73.37	68.27-68.38	68.33	72.62-72.94	72.78
Subcluster V-2- Subcluster IX-2	72.49	--	68.07	--	72.76	--
Cluster VI- Cluster VII	73.49-75.42	74.65	69.96-71.31	70.58	73.68-75.87	75.12
Cluster VI- Subcluster VII-1	75.04-75.42	75.23	70.46-71.31	70.89	75.82-75.87	75.85
Cluster VI- Subcluster VII-2	73.49	--	69.96	--	73.68	--
Cluster VI- Cluster VIII	76.99-77.43	77.24	73.56-74.20	73.83	78.92-79.18	79.04
Cluster VI- Cluster IX	72.78-73.58	73.23	68.11-68.30	68.22	72.79-73.07	
Cluster VI- Subcluster IX-1	73.33-73.58	73.45	68.17-68.18	68.18	72.79-73.07	
Cluster VI- Subcluster IX-2	72.78	--	68.30	--	72.97	--
Cluster VII- Cluster VIII	73.11-75.47	74.37	68.69-70.50	69.37	73.19-75.16	74.36
Cluster VII- Cluster IX	71.58-73.18	72.47	67.26-68.13	67.78	71.41-72.86	72.29
Cluster VII- Subcluster IX-1	72.01-73.18	72.68	67.27-68.13	67.67	71.41-72.72	72.23
Cluster VII- Subcluster IX-2	71.58-72.35	72.05	67.76-68.41	68.01	71.60-72.86	72.43
Subcluster VII-1- Subcluster VII-2	75.41-75.88	75.64	74.90-75.75	75.33	79.72-80.06	78.89
Subcluster VII-1- Cluster VIII	74.49-75.47	74.89	68.69-70.50	69.55	74.73-75.16	74.91
Subcluster VII-1- Cluster IX	72.20-73.18	72.74	67.35-68.13	67.89	72.53-72.86	72.69
Subcluster VII-1- Subcluster IX-1	72.75-73.18	72.97	67.35-68.13	67.77	72.53-72.72	72.61

Subcluster VII-1- Subcluster IX-2	72.20-72.35	72.28	67.85-68.41	68.13	72.84-72.86	72.85
Subcluster VII-2- Cluster VIII	73.11-73.60	73.35	68.83-69.21	69.02	73.19-73.36	73.27
Subcluster VII-2- Cluster IX	71.58-72.23	71.91	67.27-67.68	67.57	71.41-71.60	71.50
Subcluster VII-2- Subcluster IX-1	72.01-72.23	72.12	67.27-67.68	67.48	71.41-71.50	71.46
Subcluster VII-2- Subcluster IX-2	71.58	--	67.76	--	71.60	--
Cluster VIII- Cluster IX	72.55-73.95	73.28	67.26-68.36	67.85	72.31-72.87	72.57
Cluster VIII- Subcluster IX-1	73.36-73.95	73.51	67.60-68.36	67.88	72.41-72.87	72.63
Cluster VIII- Subcluster IX-2	72.55-73.67	72.82	67.26-68.89	67.79	72.31-72.72	72.47
Subcluster IX-1- Subcluster IX-2	76.55-76.29	76.42	76.85-77.08	76.97	81.34-81.81	81.58

Table S3 Isolation sources of the *Phyllobacteriaceae* type strains.

Strain	Isolation source	Details
Mesorhizobium sophorae ICMP 19535^T	root nodule	isolated from root nodules of *Sophora microphylla* from a river outwash fan, Pororari River, Westland, New Zealand
Mesorhizobium waitakense ICMP 19523^T	root nodule	isolated from root nodules of *Sophora microphylla* from Haast Schist rock outcrop, Waitaki River, Otago, New Zealand
Mesorhizobium sangaii DSM 100039^T	root nodule	isolated from the root nodules of *Astragalus luteolus* and *Astragalus ernestii*
Mesorhizobium kowhaii ICMP 19512^T	root nodule	isolated from root nodules of *Sophora microphylla* from an alluvial greywacke river terrace, upper Rakaia River, Canterbury, New Zealand
Mesorhizobium calcicola ICMP 19560^T	root nodule	isolated from root nodules of *Sophora longicarinata* from an alluvial limestone river terrace, Waima/Ure River, Marlborough, New Zealand
Mesorhizobium newzealandense ICMP 19545^T	root nodule	isolated from root nodules of *Sophora prostrata* from an alluvial limestone river terrace, Waima/Ure River, Marlborough, New Zealand
Mesorhizobium norvegicum 10.2.2^T	root nodule	isolated from a *Lotus corniculatus* root nodule in Norway
Mesorhizobium loti DSM 2626^T	root nodule	isolated from a root nodule on *Lotus corniculatus* (bird's-foot trefoil)
Mesorhizobium ciceri USDA 3378 ^{T}	root nodule	isolated from nodulated chickpeas grown in Spain
Mesorhizobium cantuariense ICMP 19515^T	root nodule	isolated from root nodules of *Sophora microphylla* from alluvial Greywacke river terrace, upper Rakaia River, Canterbury, New Zealand
Mesorhizobium qingshengii CGMCC 1.12097^T	root nodule	isolated from effective nodules of *Astragalus sinicus*
Mesorhizobium jarvisii ATCC 33669^T	root nodule	isolated from nodules of *Lotus corniculatus*
Mesorhizobium japonicum MAFF 303099^T	root nodule	isolated from *Lotus japonicum* in 1981 Japan
Mesorhizobium huakuii NBRC 15243^T	root nodule	isolated from root nodules of *Astragalus sinicus*
Mesorhizobium carmichaelinearum ICMP 18942^T	root nodule	isolated from *Carmichaelineae* spp. root nodules

Mesorhizobium erdmanii USDA 3471^T	root nodule	isolated from nodules of *Lotus corniculatus*
Mesorhizobium comanense 3P27G6^T	groundwater	isolated from groundwater, isolated from an artesian well connected to the thermal water basin of Comano Terme, Province of Trento, Italy.
Mesorhizobium australicum WSM2073^T	root nodule	isolated from *Biserrula pelecinus L.* in Australia
Mesorhizobium opportunistum WSM2075^T	root nodule	isolated from *Biserrula pelecinus L.* in Australia
Mesorhizobium neociceri CCANP35^T	root nodule	isolated from *C. canariense* nodules belong to a new symbiovar, for which we propose the name canariense
Mesorhizobium caraganae LMG 24397^T	root nodule	isolated from root nodules of *Caragana microphylla* growing in Beipiao city, Liaoning Province, China
Mesorhizobium prunaredense STM4891^T	root nodule	isolated from *Anthyllis vulneraria* root-nodules
Mesorhziobium muleiense CGMCC 1.11022^T	root nodule	isolated from a root nodule of *Cicer arietinum* grown in Shuangdamen villages in Qitai county (west of Mulei)
Mesorhizobium ventifaucium STM 4922^T	root nodule	isolated from root-nodules of *Anthyllis vulneraria* by trapping using soils from southern France
Mesorhizobium escarrei STM 5069^T	root nodule	isolated from root-nodules of *Anthyllis vulneraria* by trapping using soils from southern France
Mesorhizobium delmotii STM4623^T	root nodule	isolated from *Anthyllis vulneraria* root-nodules
Mesorhizobium temperatum SDW018^T	root nodule	isolated from *Astragalus adsurgens* growing in the northern regions of China
Mesorhizobium onobrychidis OM4^T	root nodule	isolated from a root nodule of *Onobrychis viciifolia*, Germany, in 2019
Mesorhizobium wenxiniae WYCCWR 10195^T	root nodule	isolated from chickpea (*Cicer arietinum L.*) in China
Mesorhizobium mediterraneum USDA 3392^T	root nodule	isolates obtained from nodules on chickpeas growing in uninoculated fields over a wide geographic range
Mesorhizobium tianshanense NBRC 102499^T	soil	isolated from an arid saline desert soil in the Xinjiang region of northwestern People's Republic of China

Mesorhizobium sanjuanii BSA136^T	root nodule	isolated from nodules of *Lotus tenuis* in the saline-alkaline lowlands of Flooding Pampa, Argentina
Mesorhizobium helmanticense CSLC115N^T	root nodule	isolated from *Lotus corniculatus* nodules in Spain
Mesorhizobium metallidurans STM2683^T	root nodule	isolates obtained from *Anthyllis vulneraria*, a metallicolous legume species, growing close to a zinc mine in the south of France
Mesorhizobium waimense ICMP19557^T	root nodule	isolated from the root nodules of *Sophora longicarinata* from an alluvial limestone river terrace, Waima/Ure River, Marlborough, New Zealand
Mesorhizobium amorphae NBRC 102496^T	root nodule	isolated from Chinese soils
Mesorhizobium plurifarium ORS 1032^T	root nodule	isolated from root nodules of *Acacia* species in Senegal
Mesorhizobium atlanticum CNPSo 3140^T	soil	isolated from soils of the Brazilian Atlantic Forest biome
Mesorhizobium hawassense AC99b^T	root nodule	isolated from root nodules of *Sesbania sesban* growing at Wondogenet, around Hawassa, the regional capital of southern Ethiopia
Mesorhizobium silamurunense CCBAU 01550^T	root nodule	isolated from root nodules of *Astragalus* species in China
Mesorhizobium tamadayense DSM 28320^T	*Astragalus adsurgens*	isolated from *Astragalus adsurgens* growing in the northern regions of China
Aquamicrobium defluvii DSM 11603^T	sludge	isolated from activated sludge of the municipial sewage treatment plant of Regensburg (Germany) with thiophene2-carboxylate as the sole source of carbon and with nitrate as the electron acceptor
Aquamicrobium lusatiense DSM 11099^T	activated sludge	isolated from an industrial wastewater-treatment plant
Mesorhizobium composti CC-YTH430^T	compost	isolated from a compost sample in Taiwan
Pseudaminobacter manganicus JH-7^T	sludge	isolated from sludge of a manganese mine
Mesorhizobium terrae NIBRBAC000500504^T	soil	isolated from soil in Jangsu, Korea
Aminobacter ciceronei DSM 17455^T	soil	isolated from agricultural soil in California fumigated with CH3Br
Aminobacter aminovorans DSM 7048^T	soil enrichments	isolated from soil enrichments containing various amines

Aminobacter carboxidus DSM 1086^{T}	soil	isolated from woodland soil in Northern Ireland
Aminobacter anthyllidis LMG 26462^{T}	root nodule	isolated from root nodule of *Anthyllis vulneraria*
Aminobacter niigataensis DSM 7050^{T}	soil	isolated as a DMF-utilizing bacterium from soil in Niigata region of Japan
Aminobacter aganoensis DSM 7051^{T}	soil	isolated as a TMAH-utilizing bacterium from soil
Pseudaminobacter salicylatoxidans DSM 6986^{T}	microbial consortium originating	isolated from a 6-aminonaphthalene-2-sulfonate-degrading microbial consortium originating from the River Elbe, Germany
Mesorhizobium soli JCM 19897^{T}	rhizosphere soil	isolated from the rhizosphere of *Robinia pseudoacacia L.* in South Korea
Pseudaminobacter arsenicus CB3^{T}	acquifer	isolated from arsenic-rich aquifers
Mesorhizobium camelthorni CCNWXJ 40-4^{T}	root nodule	isolated from a root nodule of *A. sparsifolia* in Alaer, Xinjiang Province, China
Mesorhizobium alhagi CCNWXJ12-2^{T}	root nodule	isolated from wild *Alhagi sparsifolia* in north-western China
Mesorhizobium ephedrae 6GN30^{T}	root	isolated from the roots of *Ephedra przewalskii* in Kumtag desert
Mesorhizobium albiziae DSM 21822^{T}	root nodule	isolation of the first strains from *Albizia kalkora*
Tianweitania aestuarii BSSL-BM11 ^{T}	sand	isolated from sand of coastal dunes along the Yellow Sea of the Korean peninsula
Tianweitania sediminis Z8^{T}	soil	isolated from Taklimakan Desert soil samples, and shown to be highly resistant to most β-lactam antibiotics
Tianweitania populi KCTC 42249^{T}	bark	isolated from bark tissue of *Populus×euramericana*
Nitratireductor alexandrii Z3-1^{T}	phycosphere	isolated from phycosphere microbiota of toxic marine dinoflagellate *Alexandrium tamarense* 880
Aquibium carbonis B2.3^{T}	water	isolated from coal bed water from Jincheng
Aquibium microcysteis NIBR3^{T}	*Microcystis aeruginos*a culture	isolated from a *Microcystis aeruginosa* culture

Aquibium oceanicum B7^T	sea water	isolated from deep seawater in the South China Sea
Aquamicrobium aerolatum DSM 21857^T	air	isolated from air in a duck shed
Mesorhizobium sediminum KCTC 42205^T	deep-sea sediment	isolated from deep-sea sediment collected from the Indian Ocean
Mesorhizobium microcysteis MaA-C15^T	xenic culture of *Microcystis aeruginosa*	isolated from a xenic culture of *Microcystis aeruginosa* in the Republic of Korea
Nitratireductor pacificus pht-3B^T	pyrene-degrading consortium	isolated from a pyrene-degrading consortium
Nitratireductor indicus C115^T	rude-oil-degrading consortium	isolated from a crude-oil-degrading consortium, enriched from deep-sea water of the Indian Ocean
Nitratireductor aquibiodomus JCM 21793^T	marine denitrification system	isolated from the marine denitrification system of the Montreal Biodome, Canada
Nitratireductor rhodophyticola L1-7-SE^T	marine red algae	isolated from marine red algae
Nitratireductor kimnyeongensis KACC 11904^T	dried seaweed	isolated from a dried seaweed sample collected from Kimnyeong Beach in Jeju, Republic of Korea
Chelativorans intermedius LMG 28482^T	water	isolated from water of coastal hot springs located in Taiwan and China
Chelativorans multitrophicus DSM 9103^T	sludge	isolated as aerobic EDTA-degrading bacteria from a mixture of soil extracts and activated sludge samples taken from various industrial wastewater treatment plants (Switzerland)
Chelativorans petroleitrophicus SCAU2101^T	petroleum	isolated from a mixture of oil-based drill cuttings and paddy soil
Nitratireductor aestuarii CGMCC 1.15320^T	estuary of the Jiulong River	isolated from the estuary of the Jiulong River, south-east China
Phyllobacterium zundukense Tri-48^T	root nodule	isolated from root nodules of the legume species *Oxytropis triphylla* (Pall.) Pers
Phyllobacterium sophorae CCBAU 03422^T	root nodule	isolated from root nodules of Sophora flavescens
Phyllobacterium brassicacearum STM 196^T	microaerophilic veil	isolated from a microaerophilic veil transversed by cable bacteria in freshwater sediment

Phyllobacterium bourgognense 31-25a^T	microaerophilic veil	isolated from a microaerophilic veil transversed by cable bacteria in freshwater sediment
Phyllobacterium trifolii CECT 7015^T	root nodule	isolated from a Trifolium pratense root nodule
Phyllobacterium endophyticum CECT 7949^T	root nodule	isolated from nodules of Phaseolus vulgaris
Phyllobacterium calauticae R2-JL^T	microaerophilic veil	isolated from a microaerophilic veil transversed by cable bacteria in freshwater sediment
Phyllobacterium myrsinacearum DSM 5892^T	root	isolates from sugar-beet roots
Phyllobacterium salinisoli LLAN61^T	root nodule	isolated from a *Lotus lancerottensis* root nodule in saline soil from Lanzarote
Phyllobacterium phragmitis 1N-3^T	rhizome	isolated from *Phragmites australis* rhizome in Kumtag Desert
Phyllobacterium leguminum ORS 1419^T	root nodule	isolated from root nodules of *Argyrolobium uniflorum* and *Astragalus algerianus*
Oricola indica JL-62^T	deep-seawater	isolated from deep-sea water in the southwest Indian ridge
Oricola thermophila MEBiC13590^T	tidal flat sediment	isolated from tidal flat sediment sampled at Incheon City, on the west coast of the Republic of Korea
Oricola cellulosilytica JCM 19534^T	seawater	isolated from surface seashore water of Hualien, Taiwan and subjected to polyphasic taxonomy
Oceaniradius stylonematis StC1^T	red alga	isolated from a red alga, *Stylonema cornucervi*
Roseitalea porphyridii MA7-20^T	marine red alga water	isolated from a marine red alga, *Porphyridium marinum*, in South Korea
Salaquimonas pukyongi RR3-28^T	seawater	isolated from a seawater recirculating aquaculture system in the Republic of Korea
Pseudohoeflea suaedae YC6898^T	root	isolated from the root of the halophyte *Suaeda maritima*

Table S4 The dDDH value of the species of *Mesorhizobium* with pair ANI value > 95%.

Query genome	Reference genome	ANI(%)	dDDH(%)
Mesorhizobium sophorae ICMP 19535^T	*Mesorhizobium waitakense* ICMP 19523^T	96.42	72.20±1.48
Mesorhizobium escarrei STM 5069^T	*Mesorhizobium ventifaucium* STM 4922^T	95.52	64.30±0.90
Mesorhizobium delmotii STM 4623^T	*Mesorhizobium temperatum* SDW018^T	95.14	62.70±0.98
Mesorhizobium delmotii STM 4623^T	*Mesorhizobium onobrychidis* OM4^T	95.02	62.50±1.15
